# Measurement and simulation of the relatively competitive advantages and weaknesses between economies based on bipartite graph theory

**DOI:** 10.1371/journal.pone.0197575

**Published:** 2018-05-29

**Authors:** Jun Guan, Xiaoyu Xu, Shan Wu, Lizhi Xing

**Affiliations:** College of Economics and Management, Beijing University of Technology, Beijing, China; Rutgers The State University of New Jersey, UNITED STATES

## Abstract

The input-output table is very comprehensive and detailed in describing the national economic systems with abundant economic relationships, which contain supply and demand information among various industrial sectors. The complex network, a theory, and method for measuring the structure of a complex system can depict the structural characteristics of the internal structure of the researched object by measuring the structural indicators of the social and economic systems, revealing the complex relationships between the inner hierarchies and the external economic functions. In this paper, functions of industrial sectors on the global value chain are to be distinguished with bipartite graph theory, and inter-sector competitive relationships are to be extracted through resource allocation process. Furthermore, quantitative analysis indices will be proposed under the perspective of a complex network, which will be used to bring about simulations on the variation tendencies of economies’ status in different situations of commercial intercourses. Finally, a new econophysics analytical framework of international trade is to be established.

## Introduction

Compared with firm surveys and fine industrial classification of trade, **Input-Output (IO)** tables enjoy more feasibility in measuring both standard and vertical trades. With the availability and utilization of global IO database, especially **Inter-Country Input-Output (ICIO)** tables, it is possible to construct quantitative indices to assess what degree of impact a particular sector in a country has made on the **Global Value Chain (GVC)**. This is because it better captures the international source and use of intermediate goods than any previous databases. As a result, a large number of researchers propose distinct approaches to the measurement of sectors’ function or status.

Beyond all question, IO table as a quantitative technique of economic analysis presents the interdependencies between different branches of a national or regional economy in details. Its property of being in the form of checkboard enables it to reflect the movements of products or services within the whole economic system from production consumption to distributive utilization, which is actually the formation and distribution of values respectively. The dual identities of each sector on the network as the producer and consumer at the same time, demand it not only to produce and distribute providing inputs for the other sectors but also to consume inputs from other sectors to accomplish its own fabrication. This is indeed the inner identity proposed by Karl Marx. The sectors in the IO table could be regarded as nodes while inter-industry value stream contributes to weighted and directed edges in the construction of network models.

In consideration of both availability and authority, IO table is definitely the priority-first data format to establish mathematics model, e.g., it can show flows of final and intermediate goods and services defined according to industry outputs. In addition, it is provided as a matrix, which can be directly or with minor modification adopted as complex network’s adjacency matrix, establishing weighted and directed networks.

From an empirical perspective, a handful of studies have characterized the structure of IO networks to better understanding the topology of inter-sector dependences and their repercussions on the industrial economics. For instance, Blöchl, et al. adopted TiVA database at OECD-WTO to establish 37 countries’ IO networks and derived two indicators for weighted and directed networks, which are, random walk centrality to reveal the most immediately affected nodes by a shock based on Freeman’s closeness centrality, and counting betweenness to identify the most accumulatively affected nodes based on Newman’ random walk betweenness [1]. Kagawa, et al. proposed an optimal combinatorial method to find industries with large CO2 emissions through industrial relations based on IO table, depicting environmentally important industrial clusters in Japanese automobile supply chain [2]. McNerney, et al. studied the structure of inter-industry relationships using networks of capital flows between industries in 20 national economies, and found that these networks vary around a typical structure characterized by a Weibull link weight distribution [3]. Martha, et al. investigated how economic shocks propagate and amplify through the IO network connecting industrial sectors in developed economies [4].

With the development of IO database, related researches are based not only on independent national systems but also on multi-regional even global systems, with wide adoption of WIOD as the data source. For instance, Ando measured the importance of industrial sectors under the impact of American gross output in the global IO model [5]. Antràs, et al. derived two distinct approaches to measure industry upstreamness and prove their significant impact on trade flows [6]. Cerina, et al. analyzed the subgraph structure and dynamics attributions of a global network with community detection techniques, pinpointing the key industries and economic entities with PageRank centrality and community coreness [7]. Grazzini and Spelta set up the cost effect index to testify the robustness of global IO network and the interdependency of intermediate inputs in production [8]. Johnson and Noguera combined input-output and bilateral trade data to quantify cross-border production linkages and computed bilateral trade in value added [9]. Amador and Cabral applied visualization tools and measures of network analysis on value-added trade flows in order to understand the nature and dynamics of GVC [10]. Xing, et al. established industrial complex network under the perspective of econophysics, and then analyzed the spreading effect in the form of economic shock [11], furthermore, they quantified the global industrial impact of countries on the GVC based on biased random walk process [12].

The present researches mainly mine the IO data from different aspects as an econophysics context implied in the form of networks but restricted to static analyzing endogenous variables ignoring the process of fining and refining of variables to maintain equilibrium, let alone providing measurements and advises on optimal control of the evolutionary tendency of industrial structures.

## Methodology

### Bipartite graph

Bipartite graph, or bigraph, divides the vertex set of a simple graph *G* as two nonempty sets *V*_1_ and *V*_2_ with no intersection in between. Letting the two nodes relevant to each edge in *G* belonging to *V*_1_ and *V*_2_ respectively, it can be noted as *G* = (*V*_1_,*V*_2_,*E*), in which *V*_1_ and *V*_2_ are the bisections of *G* with *E* as the set of edges. For the bipartite graph *G*, if |*V*_1_| = *m* and |*V*_2_| = *n*, and there exists an edge between two vertexes, when and only when one of the nodes belongs to *V*_1_ and the other belongs to *V*_2_, the graph can be referred to as the complete bipartite graph of vertexes *m* and *n*, noted as *K*_*m*,*n*_. The bipartite graph has a wide application in complex network analysis, including cooperation and competition networks (mainly dealt with through affiliation networks), for either cooperation or competition is the common existence in social networks consisting of people of units of people. Networks of scientists (authors and papers), patents declaration (patents and holders), commodity (goods and consumers), public transportation (routes and stops) etc. can all be clarified as affiliation networks to be digested as bipartite graphs in the manner of two-mode networks. Two kinds of vertexes exist in this kind of networks, one is that of participants and the other of objects.

**Fig 1:**
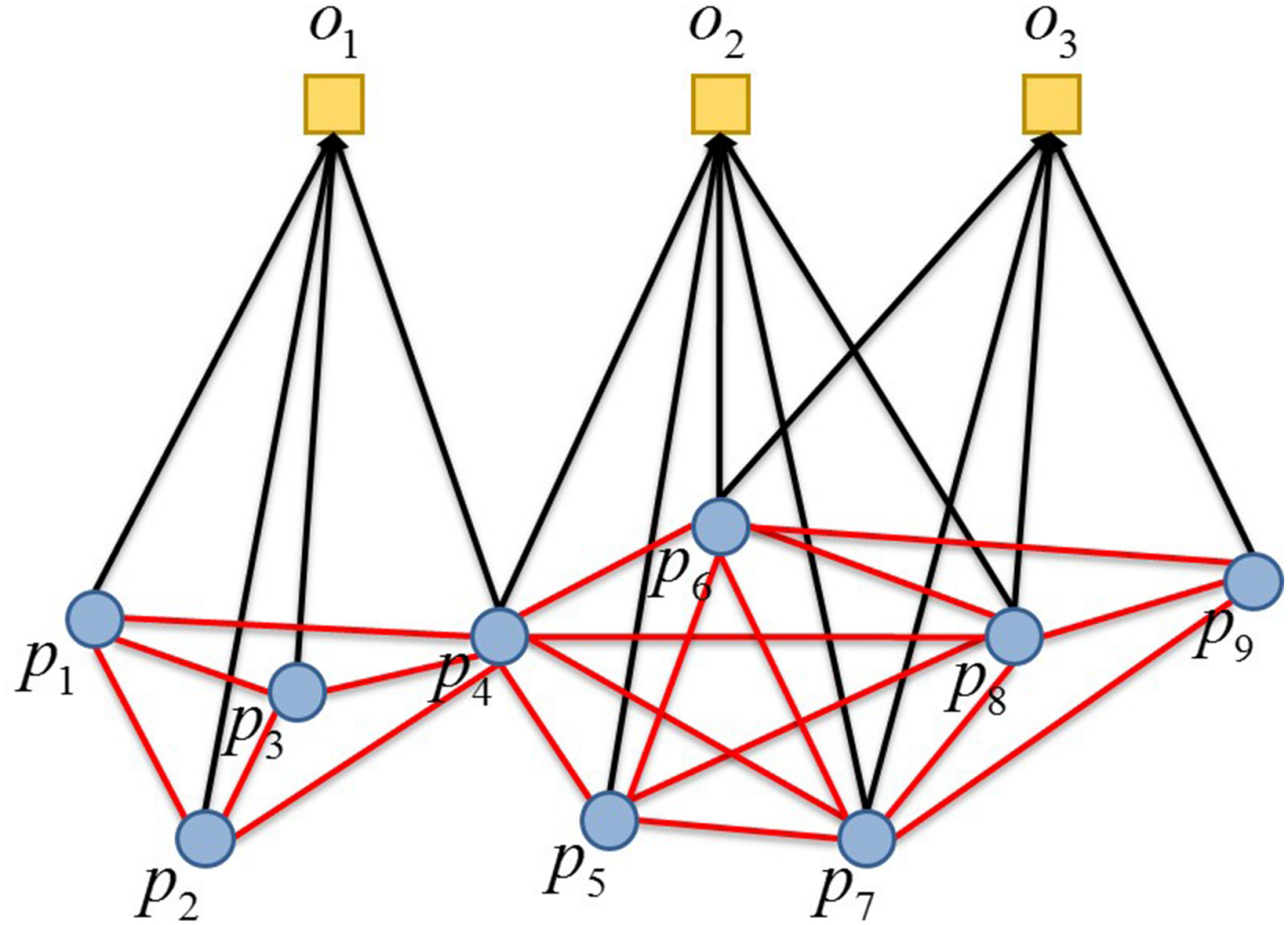
A two-mode network and its projection.

For the cooperation or competition, focusing on the interaction among vertexes of the same kind is the practical target in building two-mode networks, It is more than common to project the networks onto one kind of the vertexes (often those of participants) reaching a one-mode network. Through this projection, edges have been granted the property to reflect the relationship of cooperation or competition on the same object by two participants. This one-mode network obtained is called the complete subgraph of the object, as shown in Fig 1.

In Fig 1, the squares in the up are the objects, while the lower circles are the participants, and the edges in black belong to the two-mode networks, while those in red to the one-mode networks contributing to complete subgraphs, as each of the edges is gained through projection of two edges in the two-mode networks.

Most commonly, the one-mode network obtained through projection has edges of no weights. Yet recent researches on two-mode networks find that these weights could be gained through the definition of co-occurrences, which is the counting of the fellowship of two participants in the same object, say the number of papers of two scientists as co-authors. Newman made an extension of the process on scientist network [13], and Padrón believed that this modeling process could bring distinctive simulation on the potential cooperation or competition relationship [14].

### Resource Allocation Process

In order to minimize the information loss in the process of projection of two-mode networks, and also to take the scarcity of vertex into consideration, the **Resource Allocation Process (RAP)** is adopted in this paper as the algorithm of projection [15].

Let *V*_1_ in *G* = (*V*_1_,*V*_2_,*E*) as the vertex set of participants, represented as *P*, and *V*_2_ as that of the objects, represented as *O*, then a bipartite graph *G* = (*P*,*O*,*E*) is reached, in which, *E* is the set of edges, while vertexes in sets *P* and *O* are (*p*_1_,*p*_2_,⋯,*p*_*n*_) and (*o*_1_,*o*_2_,⋯,*o*_*m*_) respectively. The initial resource allocated to the *i* th participant is *f*(*p*_*i*_)≥0.

First of all, all the resources of *P* flow in the direction of *O*, and the resource allocation of the *l* th vertex in *O* is

$$f(o_{l})=\sum_{i=1}^{n}\frac{a_{il}f(p_{i})}{k(p_{i})}$$ (1)

where, *k*(*p*_*i*_) is the degree of *p*_*i*_, *a*_*il*_ is a *n*×*m* matrix:

$$a_{ij}=\{\begin{array}{cc} 1 & p_{i}o_{l}\in E\\ 0 & otherwise \end{array}$$ (2)

**Fig 2:**
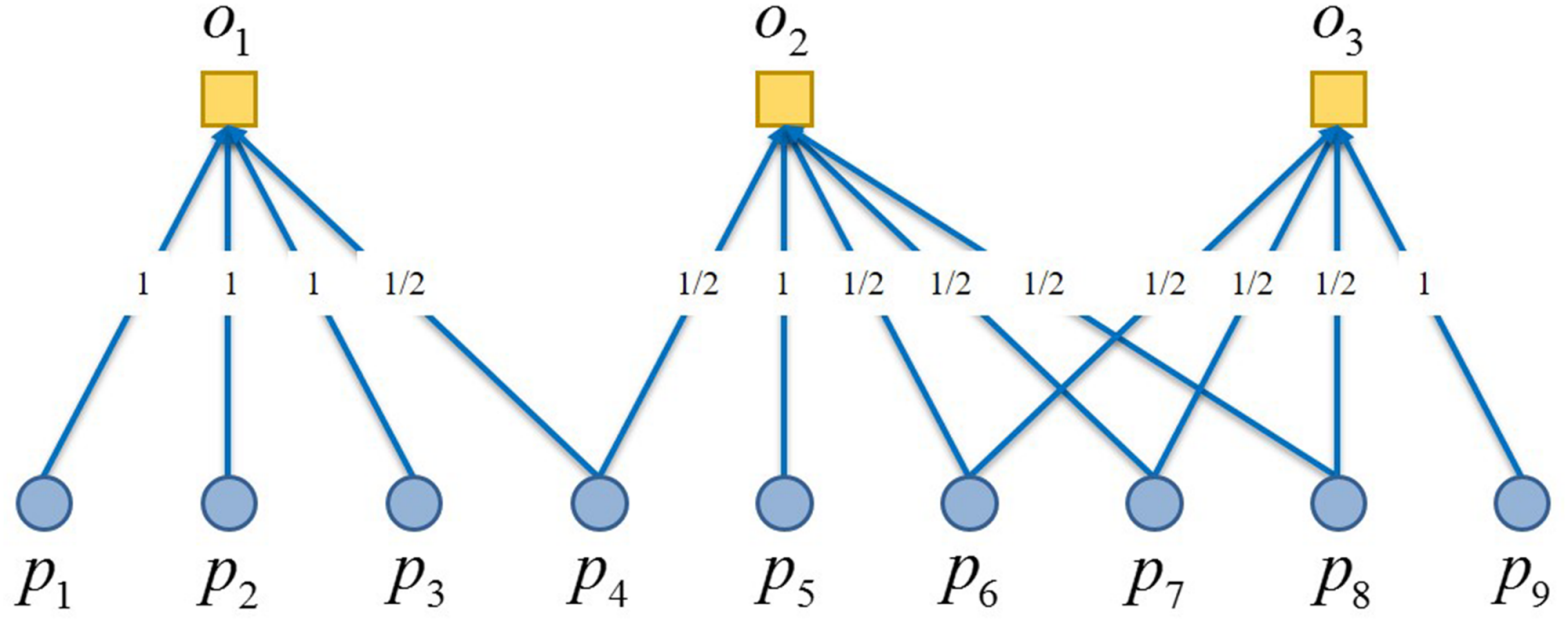
The resources owned by participants are equally distributed to objects.

The resource allocation process of *P*→*O* is shown in Fig 2.

With all the resource flown back to set *P*, the final distribution to vertex *p*_*i*_ is

$$f^{\prime }\left(p_{i}\right)=\sum_{l=1}^{m}\frac{a_{il}f\left(o_{l}\right)}{k\left(o_{l}\right)}=\sum_{l=1}^{m}\frac{a_{il}}{k\left(o_{l}\right)}\sum_{j=1}^{n}\frac{a_{jl}f\left(p_{j}\right)}{k\left(p_{j}\right)}$$ (3)

This formula could be rewritten as:

$$f^{\prime }\left(p_{i}\right)=\sum_{j=1}^{n}w_{ij}f\left(p_{j}\right)$$ (4)

**Fig 3:**
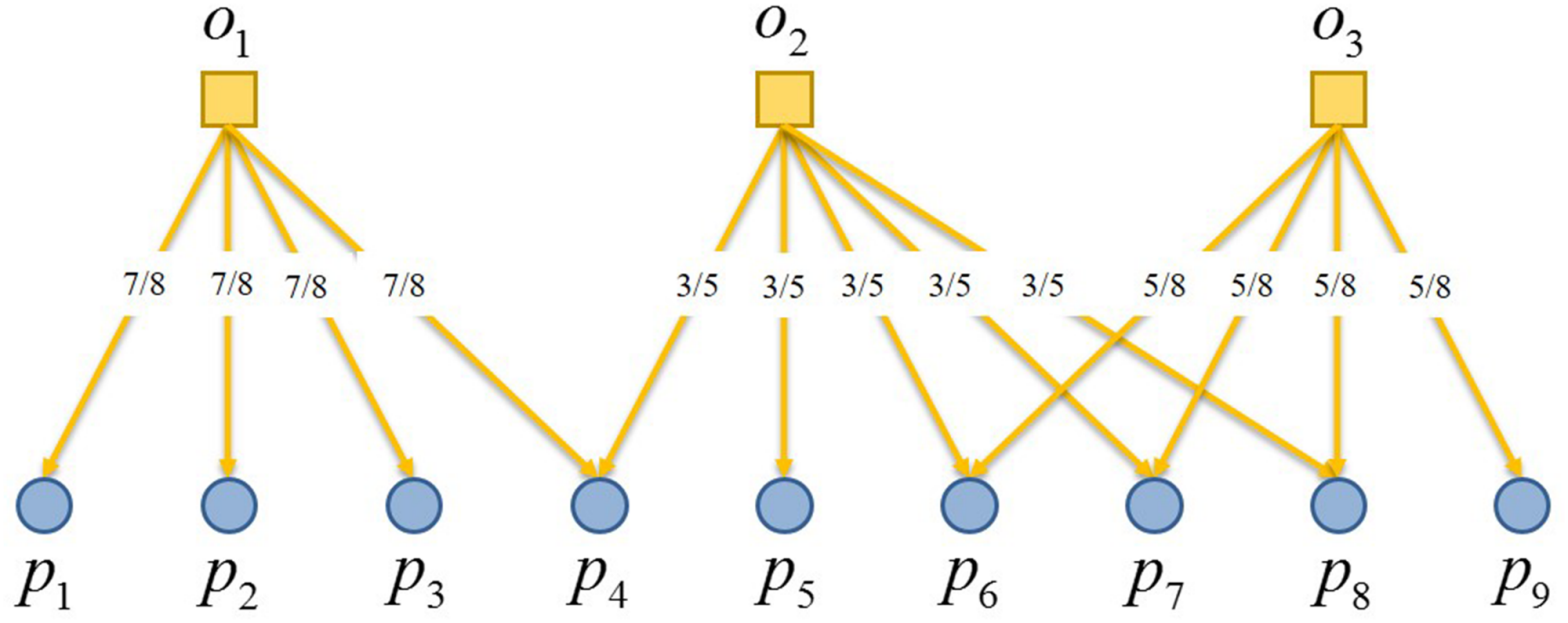
The resources owned by objects are equally distributed to participants.

The resource allocation process of *O*→*P* is shown in Fig 3.

**Fig 4:**
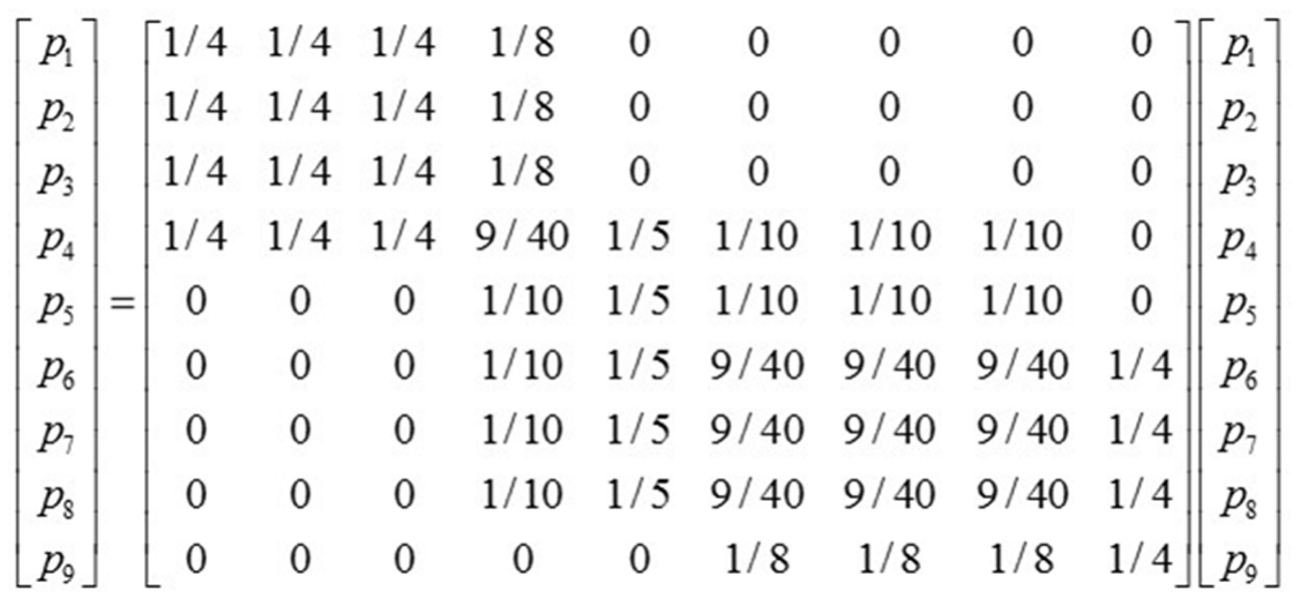
Linear relationship between objects’ resources allocation and complete object subgraph by RAP.

The $w_{ij}^{P}$ in Eq (4) could be written as:

$$w_{ij}^{P}=\frac{1}{k\left(p_{j}\right)}\sum_{l=1}^{m}\frac{a_{il}a_{jl}}{k\left(o_{l}\right)}$$ (5)

where, $w_{ij}^{P}$ is the relationship strength produced in the two resource allocation processes between *p*_*i*_ and *p*_*j*_, so the adjacency matrix $W^{P}={\left\{w_{ij}^{P}\right\}}_{n\times n}$ can be constructed for this complete object subgraph through RAP, as shown in Fig 4.

**Fig 5:**
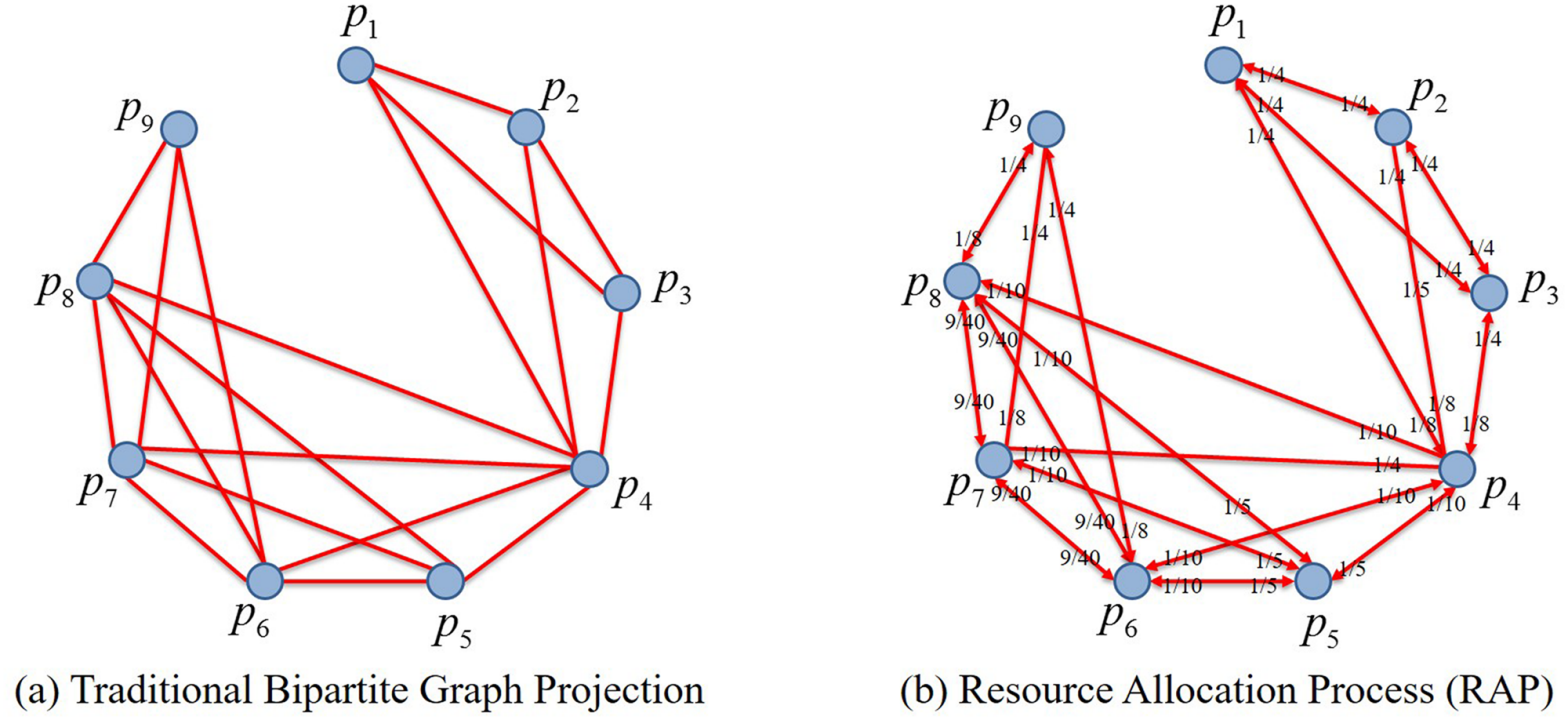
Comparison between traditional bipartite graph projection and resource allocation process.

In conclusion, the core of RAP is to have resources distributed to each participant and object in the network, with $w_{ij}^{P}$ represents the proportion of resources distributed to the participant *j* through the object from the participant *i*. Say each participant equally distribute its resources to the objects it will take a part in, and then each object will redistribute resources it received back to its participants equally through the edges of the bipartite graph. So there lies the fundamental difference between RAP and traditional bipartite graph projection, as shown in Fig 5.

RAP shares the following three characteristics:

1. The adjacency matrix *W*^*P*^ of the complete object subgraph is asymmetric, and $w_{ij}^{P}/k\left(p_{j}\right)=w_{ji}^{P}/k\left(p_{i}\right)$.
2. As two participants take parts in the same object for multiple times, their relationship strength goes from intimacy to saturation rapidly.
3. The relationship strength between two participants is decided by not only the number of times they jointly take parts in the same object but also the number of participants at the same time of the very object.

Further extension of RAP can also be made to the condition of weighted edges in bipartite graphs, when resources are no longer distributed equally, with the weight representing the degree of membership of participant’s vertex to the object’s. The formula is:

$$w_{ij}^{P}=\frac{1}{s\left(p_{j}\right)}\sum_{l=1}^{m}\frac{w_{il}w_{jl}}{s\left(o_{l}\right)}$$ (6)

where, *s*(*p*_*j*_) is the weight of participant node *p*_*j*_, $s\left(p_{j}\right)=\sum_{l=1}^{m}w_{jl}$, and *s*(*o*_*l*_) is the weight of object node *o*_*l*_, $s\left(o_{l}\right)=\sum_{i=1}^{n}w_{il}$. *w*_*il*_ and *w*_*jl*_ are the weights on edges connecting *p*_*i*_ and *p*_*j*_ with *o*_*l*_ respectively.

Thus, RAP reflects the scarcity of resources of object nodes, and at the same time the limitation of resources taken by participant nodes from object nodes, enabling the complete object subgraph obtained through projection giving a clear indication on the cooperation or competition relationship between participants.

### IO analysis using bipartite graph

IO table is good at presenting the complicated interdependent relationship among various industrial sectors from a global prospective, with a clear embodiment of the amount of resources one sector may gain from its upper-stream sectors. So researches on IO table mainly take the advantage of depicting the topological structure of the economic system through the measurements on intermediate products as an indication of input and output relationship, so as to bring lights on analysis on the rules of value flows and industrial structural features. Making observation from the prospective of bipartite graphs on the rows of IO table indicating the supply from upper to lower-stream industrial sectors and columns indicating on the demand from lower to upper ones, it is obvious that IO table is proficient in showing the cooperation or competition relationship among different industrial sectors. Yet no coopetition relationship among industrial sectors could be reflected through direct structural measurement on the IO network, with adequate matrix transformations to be introduced for this goal.

Porter proposed the nature of competitive strategy was building the relations between the corporation and its environment [16]. Actually, if there exists more than one supplier or consumer for one single industrial sector, cooperation or competition shows up, for the scarcity of resources provides a limitation to any flow of intermediate form upper to lower stream sectors. It is defined and extended by Porter’s competitive advantage, rather than the conceptual “competition” mentioned from economics. Traditional IO theory uses direct consumption and complete consumption coefficients to present this scarcity, with influence and reaction coefficients presenting the relations between one industrial sector and its environment. Yet it still bears the shortcoming that its focuses are restricted to the linear technical-economic relationships among different industrial sectors and between the gross output and final usage, neglecting the scarcity of productive resources as constraints on cooperation and competition relationships. So this paper devotes to set up modeling analysis with bipartite graph theory on the IO data, aiming at restoring the competition relationship between lower stream industrial sectors from the pro

To be noted that the flows in bipartite graphs are in the direction from the participant nodes to the object nodes, but the IO networks experience the complete contradiction of flowing from the upper stream sectors to the lower ones at the mercy of showing the value flows. Although the following analysis is based on the complete object subgraph of a bipartite graph, the flows on edges are in the opposite direction with those of the bipartite graphs.

**Fig 6:**
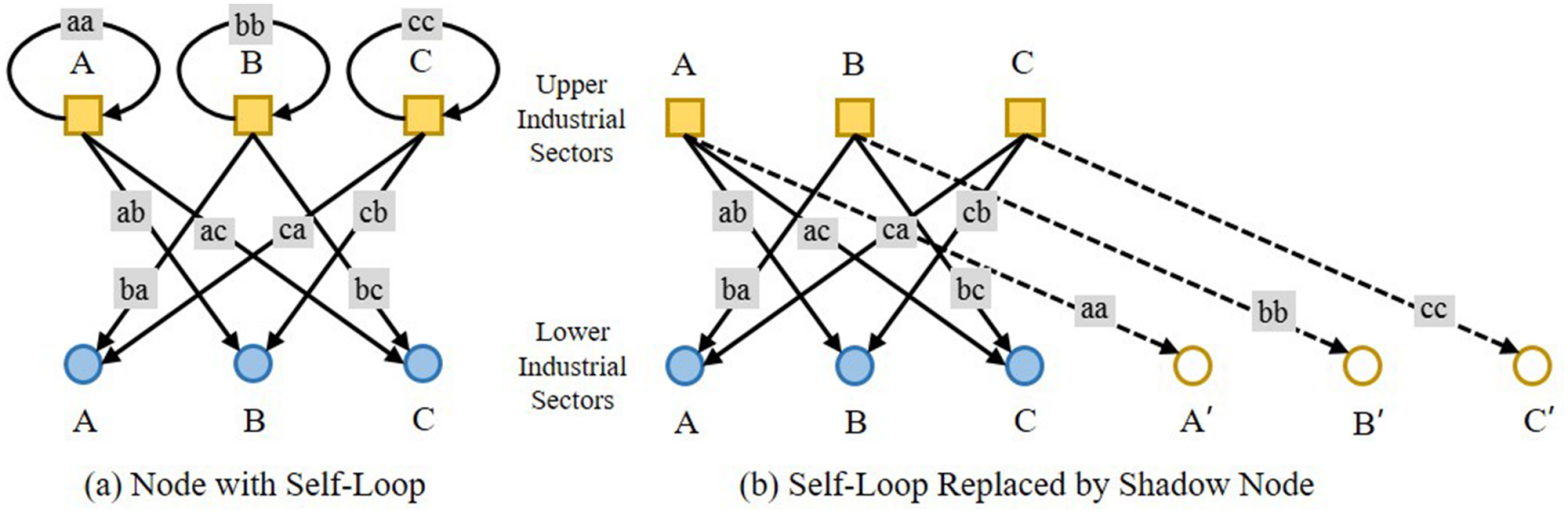
The bipartite graph of upper and lower industrial sectors and self-loop.

The upper stream sector in the IO table could be referred to as the object nodes in the bipartite graph, while the lower one as the participant nodes, as shown in Fig 6(a). The A, B, and C of squares indicate the upper stream industrial sectors, while those of circles the lower stream ones, and *ab*, *ba*, *ac*, *ca*, *bc* and *cb* are the IO values between the upper and lower industrial sectors, or in another word, the weights on the edges of the bipartite graph. *aa*, *bb* and *cc* indicate the input of one industrial sector’s own products into itself, or the weights on the self-loop of one industrial sector.

While constructing a bipartite graph using IO data, the most important is to have all the data and endowed information formatted to be applicable for the two-mode network. So long as there is no conception of self-loop in the bipartite graph theory, the concept of shadow node is here introduced to simulate the condition of self-loop of the nodes, as shown in Fig 6(b), in which the self-loops of upper stream industrial sectors A, B and C are spread into virtual edges connecting to the shadow sectors of A’, B’ and C’. While this bipartite graph is projected to the lower stream industrial sectors, the virtual edges function as connections between the upper and lower stream industrial sectors, so as to have the information of self-loop of any industrial sector reserved.

**Fig 7:**
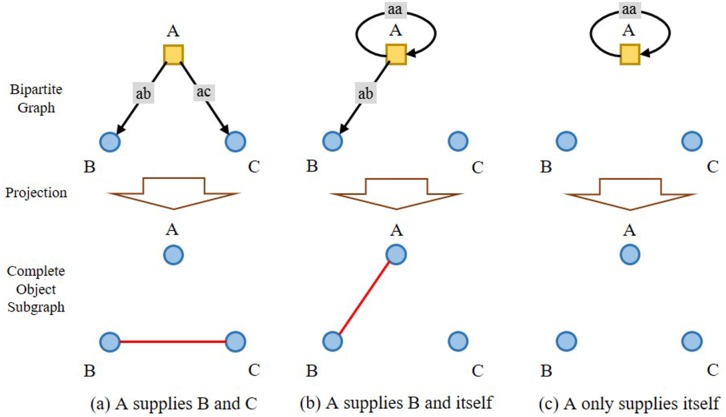
Three fundamental conditions of production resources allocation among industrial sectors on an IO network.

Three fundamental conditions exist for the production resources allocation among various industrial sectors on the IO networks, as shown in Fig 7.

1. A provides production resources simultaneously to B and C, with their IO values giving a quantitative measurement to the process, as shown in Fig 7(a). Competition shows up between B and C, and it gets intensified if they share more upper stream providers. One single competition strength can be defined in the course of the projection, and the multiple competition strengths will be definitely larger than the single one because that it will be intensified under the condition of existence of more than one A for both B and C.
2. A provides production resources simultaneously to B and as feedback to itself, as shown in Fig 7(b). The single competition strength is relevant to *ab* and *aa*, but there will not be multiple competition between A and B, for A cannot be multiplied in this case.
3. A provides production resources only to itself as feedback, as shown in Fig 7(c), and there exists no competition.

So, if any industrial sector enjoys with any other sector more than one upper stream industrial sector as production resources provider, there exist edges in the complete object subgraph depicting the competition relationship. The above discussed three fundamental conditions co-exist interdependently in the IO networks, making hurdles for the traditional methods on a reenactment of the direct or indirect competitions among industrial sectors. RAP, under this scenario, is adopted to implement projection from the upper stream industrial sectors (objects) to the lower ones (participants).

## Underlying database

**Fig 8:**
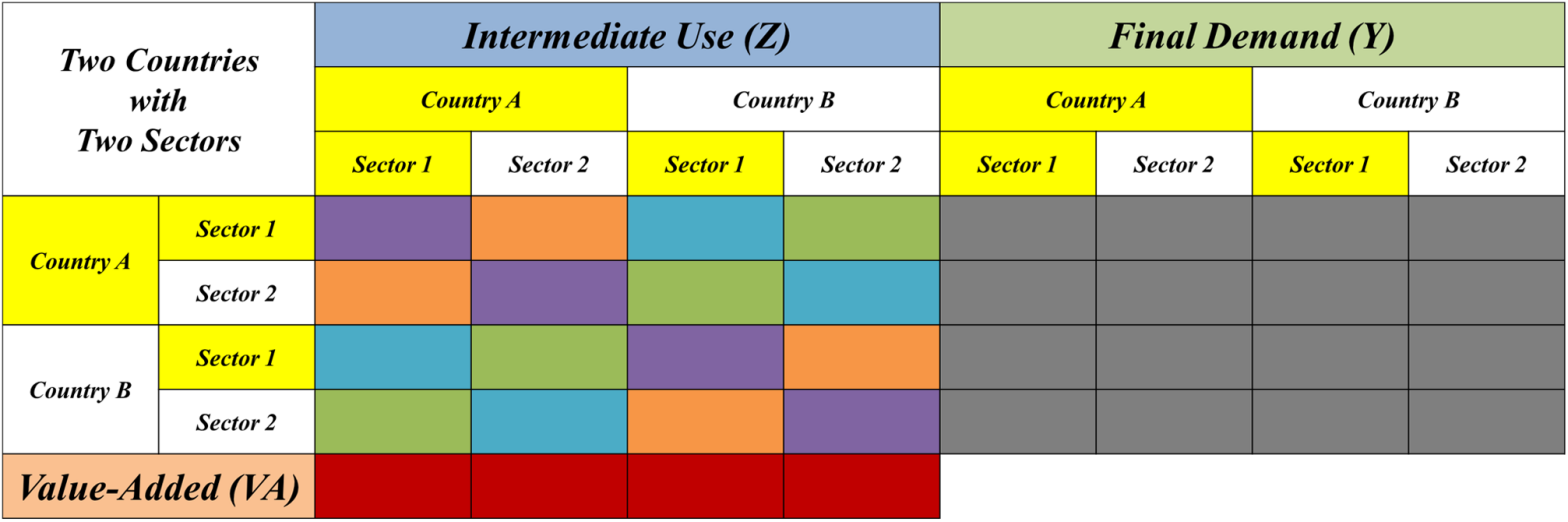
Layout of ICIO table.

With the advent of ICIO databases, it is theoretically and empirically possible to analyze the GVC, which is composed of abundant international and domestic industrial value chains, because such tables provide globally consistent bilateral trade flows and allow comparison of production networks in different regions. The layout of a normal ICIO table is shown in Fig 8, and we took the region of inter-country inter-industry use and supply as modeling data source in this paper, i.e., the yellow part of the table, in order to depict the transferring process of intermediate goods under the perspective of complex network.

There are 7 main ICIO databases available presently: World Input-Output Database (WIOD), OECD-WTO Database on Trade in Value-Added (TiVA), Eora Multi-Region Input-Output Table Database (MRIO), Global Trade Analysis Program (GTAP), Asian International Input-Output (AIO), Asian Development Bank Multi-Regional Input-Output Tables (ADB-MRIO) and Externality Data and Input-Output Tools for Policy Analysis (EXIOPOL).

As a sort of value-type IO, WIOD was chosen as the underlying database, because it provides time-series data of 40 independent countries/regions (with Taiwan as an inalienable part of Chinese territory) and the rest of world (RoW), covering the period from 1995 to 2011. These tables have been constructed in a clear conceptual framework on the basis of officially published IO tables in conjunction with national accounts and international trade statistics [17].

Furthermore, there are three different types of data in WIOD, including World Input-Output Table (WIOT), Regional Input-Output Table (RIOT) and National Input-Output Table (NIOT), with all of which as value-type IO data.

In this paper, more attention is paid on the theoretical rather than empirical analysis, so RIOT of WIOD was further chosen to establish industrial complex networks and analyze the GVC constituted by worldwide industrial chains. RIOT includes 6 economic entities, which are Eurozone, Other EU (non-Eurozone), North American Free Trade Agreement, China, East Asia and BRIIAT (an economic union) as shown in Table 1, as well as RoW (detailed names and abbreviations of sectors in RIOT of WIOD are in S1 Table).

**Table 1:** Economic entities in RIOT of WIOD.

Regions	Abbr.	Countries
Eurozone	EURO	Austria (AUT), Belgium (BEL), Cyprus (CYP), Germany (DEU), Spain (ESP), Estonia (EST), Finland (FIN), France (FRA), Hellenic (GRC) Ireland (IRL), Italy (ITA), Lithuania (LTU) Luxembourg (LUX), Malta (MLT), Netherlands (NLD), Portugal (PRT), Slovak (SVK), Slovenia (SVN)
Other EU	OEURO	Bulgaria (BGR), Czech (CZE), Denmark (DNK) England (GBR), Hungary (HUN), Latvia (LVA), Poland (POL) Romania (ROU), Sweden (SWE)
North American Free Trade Agreement	NAFTA	Canada (CAN), Mexico (MEX), America (USA)
China	CHN	China (CHN)
East Asia	EASIA	Japan (JPN), Korea (KOR), Taiwan (TWN)
BRIIAT	BRIIAT	Australia (AUS), Brazil (BRA), Indonesia (IDN), India (IND), Russia (RUS), Turkey (TUR)

Notice: Latvia joined the Eurozone in 2014, and Taiwan Province is an inalienable part of Chinese territory.

## Modeling

The RIOT data of WIOD database is chosen as the source of modeling data for this paper. There include two steps in the proposed modeling process. The first is to set up the intermediate input matrix to portray the topological structure of the global economic system with IO table. The second is to mine the direct and indirect competition relationship among industrial sectors of economic entities based on bipartite graph theory and RAP method.

### GIVCN-RIOT model

In order to establish an industrial complex network, a sector within a region is to be considered as a node, and the inter-industry IO relationship as a tie, whose weight represents the sale and purchase relationships between producers and consumers. Thus, a graph *G* = (*V*,*E*,*W*) containing *n* nodes is created, representing sectors within a nation or region, denoted as a node set *V*. Pairs of nodes are linked by ties reflecting their interdependencies, constituting an asymmetric tie set *E*. However, in valued graphs, a set *E* can actually be replaced by weight set *W*, which can be extracted from the region of inter-country inter-industry use and supply in RIOT.

**Fig 9:**
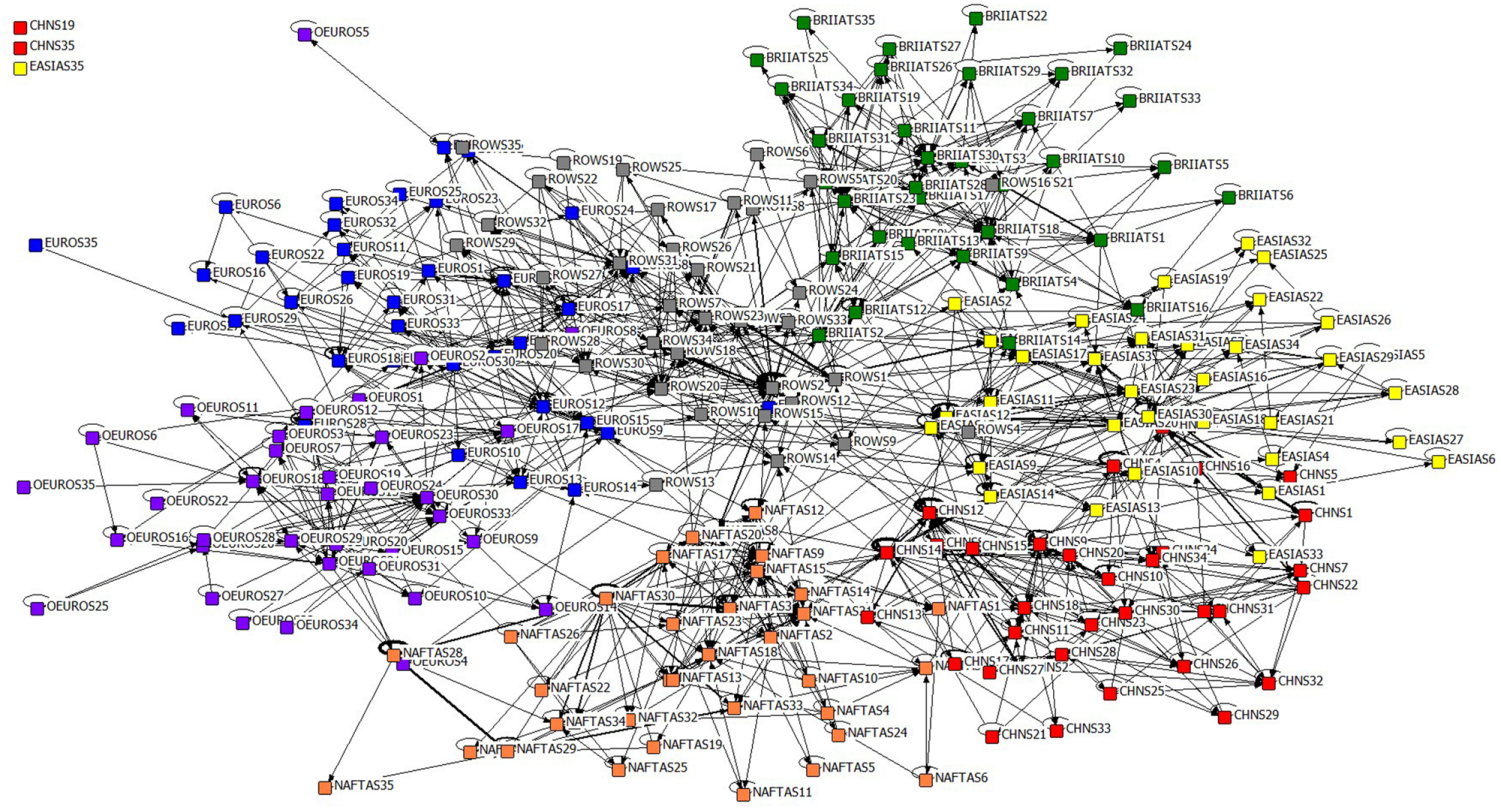
GIVCN-RIOT-2011 model.

The model built here is named as **Global Industrial Value Chain Network (GIVCN)**, since its purpose is to reflect how economic shocks propagated and amplified along GVC, as well as to what extent the industrial impact is to be created on the inter-country level. Adjacency matrices of 17 GIVCN-RIOT models can be completely downloaded from the website WIOD. For simplicity, the topological structure of GIVCN-RIOT-2011 is shown in Fig 9 after deleting weak industrial relevance based on revised Floyd algorithm (so as the other network models in this paper).

Labels of nodes in Fig 9 are constituted of two parts: abbreviation of the economic entity and a serial number of the industrial sector. Nodes of the same color indicate that they are different industrial sectors belonging to the same economic entity. It is noticed that three industrial sectors breaks away from the maximum conjunction branch of the GIVCN-RIOT-2011 model, including China’s “Sale, Maintenance and Repair of Motor Vehicles and Motorcycles; Retail Sale of Fuel” and “Private Households with Employed Persons” (CHNS19 and CHNS35), East Asia’s “Private Households with Employed Persons” (EASIAS35) due to lack of statistic in RIOT database. GIVCN-RIOT model has the following three properties:

1. GIVCN-RIOT model is a directed and weight network, in which the nodes play the roles of upper and lower stream industrial sectors simultaneously on the GVC. The ties between paired nodes and weights on them represent the IO relationship between industrial sectors in the form of directions and quantities of the value flows
2. The density of nodes in subsets is obviously higher than that of nodes of different subsets in GIVCN-RIOT model. This indicates that industrial sectors of the same country or region enjoy denser IO relationship than those of different countries or regions, proving that most commerce on the GVC happens in different economic entities.
3. There is an abundant existence of self-loop with nodes in GIVCN-RIOT model, and even with very large weights on the edges, stating clearly that the consumption of its own products as the intermediate for production is more than common for many industrial sectors.

### GIVCN-RIOT-BIPARTITE model

With its data structure enclosing the competition and cooperative relationship among industrial sectors, GIVCN-RIOT model reveals the mechanism of creation, distribution, transfer, and value-addition of value on the GVC. The classical IO analysis adopts the direct consumption and complete consumption coefficients matrices to show the direct and indirect technical-economic relationships among industrial sectors, before using influence and reaction coefficients to measure the pulling effect and demand intensity of one sector on the other. But no existing research has brought researches on the competitive relations to the level of industries, for there lies the difficulty of distinguishing the functional roles of any industry in outputting or consuming the intermediates. Therefore, a two-mode network is here to be introduced to open the lid off the hidden competitive relationship, either direct or indirect, in the GIVCN-RIOT model, with the following assumptions:

1. All the upper-stream industrial sectors contribute to the set of object nodes *O*, the lower-stream industrial sectors constitute to the set of nodes of the first-category participants *P*′, and the shadow nodes obtained through self-loop make the composition of the set of nodes of the second-category participants *P*″. Therefore, the set of nodes of all participants makes *P* = *P*′ ∪ *P*″. For the industrial sectors in IO tables show up in the status of both upper and lower stream simultaneously, and also there are self-loops for all the industrial sectors, |*O*| = |*P*′| = |*P*″| = *N*, |*P*| = 2*N*.
2. Edges are directed from the upper-stream industrial sectors to the lower-stream ones, making known to the flowing directions of the intermediates. Edges between the two categories of nodes form the edges set *E*′. Self-loop of each node reflects the industrial sector’s consumption of part of its own output as input, so those industrial sectors with self-loops are considered to be the upper or lower stream industrial sectors of themselves in this paper, say the object node has an edge to its shadow node as *E*′, then *E* = *E*′ ∪ *E*″.
3. Similar assumptions are laid to the sets of weights. The set of weights between the upper and lower industrial sectors are *W*′ and those of industrial sectors’ consumption of their own output as input are *W*″. The weight set of the whole network is *W* = *W*′ ∪ *W*″. Among *N*−1 competitors, the lower-stream industrial sector *i* obtains the number of ${w^{\prime }}_{li}$ intermediates from its upper-stream industrial sector *l*, while the amount of self-consumption of its own output is noted as ${w^{\prime \prime }}_{li}$ (when *l* = *i*, indicating the upper and lower stream industrial sectors are practically the same one.)

**Fig 10:**
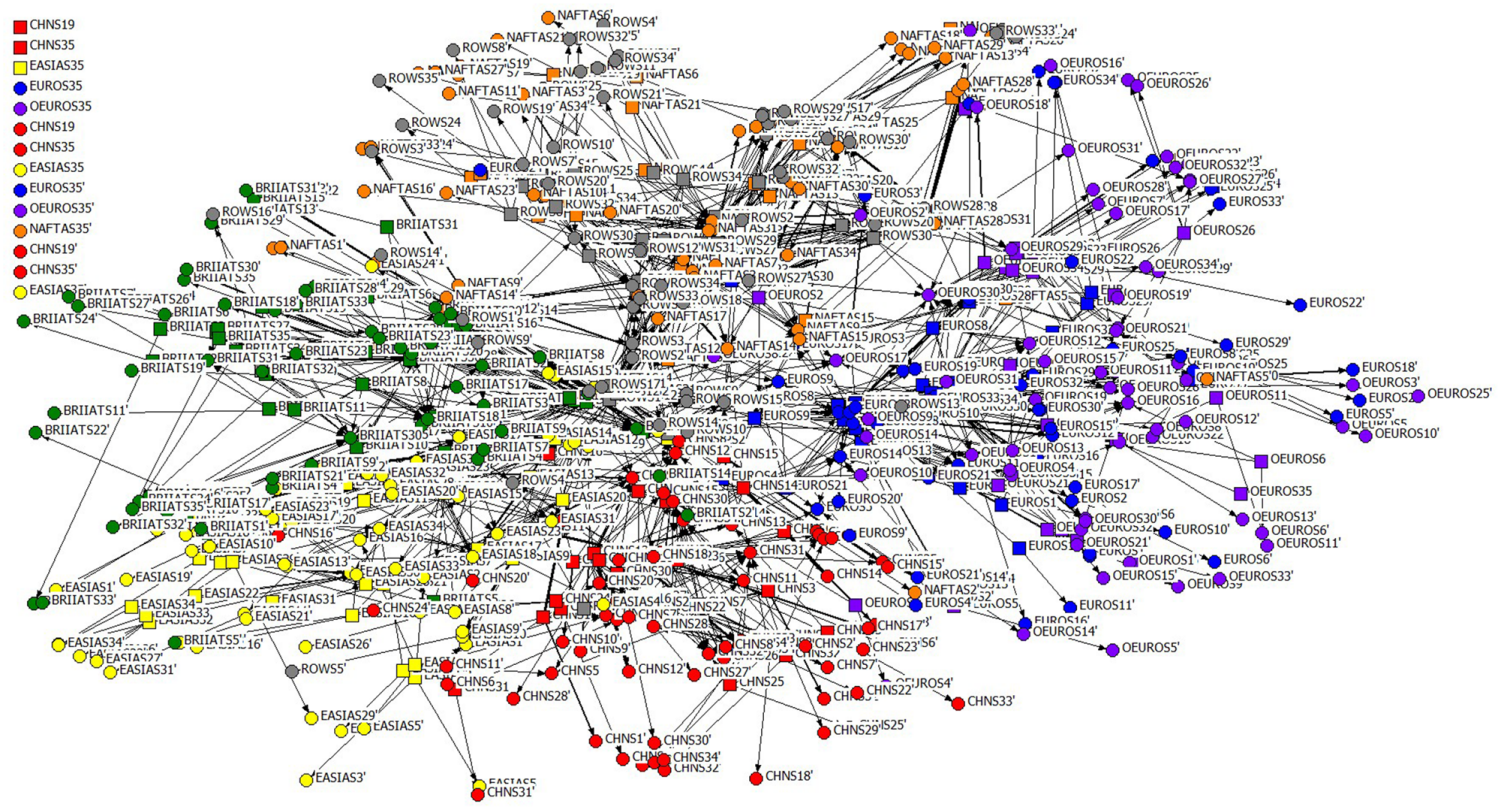
GIVCN-RIOT-BIPARTITE-2011 model.

Based on the above assumptions, the GIVCN-RIOT model has turned from a simple graph *G* = (*V*,*E*,*W*) to a bipartite graph *G* = (*O*, *P*′, *P*″, *E*′, *E*″, *W*′, *W*″), which is named GIVCN-RIOT-BIPARTITE model, as shown in Fig 10.

Nodes in the shapes of rectangular in Fig 10 reflect the set of object nodes *O* composed of upper-stream industrial sectors, and those in shapes of dots for the set of object nodes *P* of the lower-steam ones. Distinguishment has also been made between the two categories of participant nodes, e.g. NAFTA35’ represents the self-consumption of its own output by NAFTA “Private Households with Employed Persons”. Edges exist only between nodes of different categories in the GIVCN-RIOT-BIPARTITE model, and self-loops in GIVCN-RIOT models are the edges between the nodes and their own shadow nodes.

### GIRCN-RIOT model

With the global economic system under explanation by GIVCN-RIOT-BIPARTITE model, the lower-stream industrial sectors consume the limited output by the upper-stream ones, proving the scarcity of production resources. When several lower-stream industrial sectors enjoy the same upper-stream one as the feeder of production resources, the scarcity is transferred into competition relations among the lower-stream industrial sectors. Under the help of projection algorithm RAP, the competitive relations implied in the GIVCN-RIOT model can be shown by its complete object subgraph, and the formula of projection is as follow:

$$w_{ij}^{P}=\{\begin{array}{cc} \frac{1}{w_{j}}\sum_{l=1}^{N}\frac{w_{li}w_{lj}}{w_{l}} & i\neq j\\ 0 & i=j \end{array}$$ (7)

where, *w*_*l*_ is the gross output of upper-steam industrial sector *l*, and it is numerically equal to the output weight of industrial sector *l* in the GIVCN-RIOT-BIPARTITE model, say $w_{l}=S^{OUT}\left(l\right)=\sum_{i=1}^{N}w_{li}$, *i*,*l* ∈ {1,2,⋯,*N*}. $w_{ij}^{P}$ is the competitive strength of the industrial sector *i* against *j*, both of lower-stream status, when they both belong to the lower-stream industrial sectors competing for intermediates from a common upper-stream industrial sector for production resources, contributing to the edge weights set $W^{P}=\left\{w_{ij}^{P}\right\}$, *i*,*j* ∈ {1,2,⋯,*N*}. The $e_{ij}^{P}$ connecting node *v*_*i*_ to *v*_*j*_ in the complete object subgraph depicts how sector *i* obtaining intermediate from its upper-stream sectors has influenced the benefit of sector *j*, with the weight of edge $w_{ij}^{P}$ indicating the degree of the influence. Those on the diagonal line of matrix *W*^*P*^ are set to be zero, for it is the competitive relations among various industrial sectors to be analyzed in this paper.

**Fig 11:**
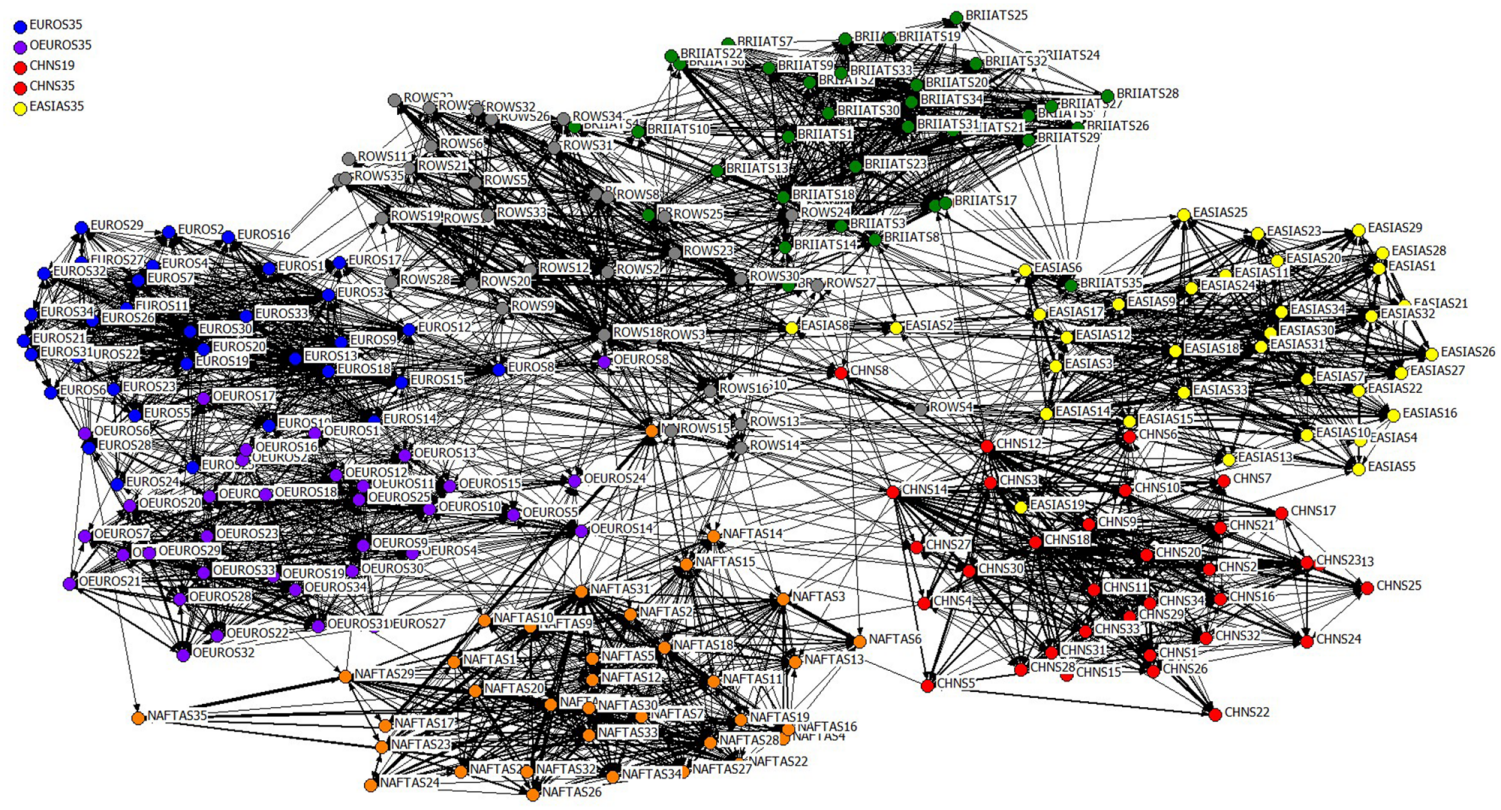
GIRCN-RIOT-2011 model.

Till now, the projection of the complete object subgraph $G^{P}=\left({V^{\prime }}_{2},E^{P},W^{P}\right)$ analyzing the direct and indirect competitive relations among industrial sectors of the global economic system has been constructed, and it is referred to as the **Global Industrial Resource Competition Network (GIRCN)** hereafter. GIRCN-RIOT is a sort of weighted and directed one-mode network without any self-loop of any node. Fig 11 shows the topological structure of GIRCN-RIOT-2011 (simplified with the modified Floyd algorithm), in which the widths of the edges are proportional to their weight.

It is easy to notice that there exist obvious agglomerations in the model of GIRCN-RIOT-2011 and competitions mainly exist among industrial sectors with economic entities. The integration between the Euro zone countries and other EU ones are comparatively more intensive. Compared to the GIVCN-RIOT-2011 model, the “Private Households with Employed Persons” of the Euro and non-Euro regions (EURO35 and OEURO35) get detached from the maximum conjunction branch, for these two sectors are only the source nodes at the front of the network, having no competition with any of the other industrial sectors. Moreover, although all the shadow nodes belong to the set of participant nodes, no connecting edge could be found for this sort of nodes after the projection. This paper emphasizes on the competitive relations among industrial sectors, so the connecting edges between the shadow nodes and original ones can also be ignored, as an elimination of the influence of one industrial sector’s consumption of its own output upon its own benefit. Thus there are no shadow nodes in GIRCN-RIOT-2011 model.

## Measurement

### CAI and CWI

The edge weight set *W*^*P*^ of GIRCN-RIOT indicates the direct and indirect competitive relations among industrial sectors. It is to be noticed that this competitive relation is directed, e.g. $w_{ij}^{P}$ is the competitive strength of industrial sector *i* against *j*, while $w_{ji}^{P}$ is that of the opposite. So it is defined in this paper that the summation of the competitive strengths of an industrial sector to be its **Competitive Advantage Index (CAI)**, and the summation of strengths of one industrial sector to be competing against as **Competitive Weakness Index (CWI)**.

Judged from the prospective of complex networks, CAI and CWI are the out-strengths *S*^*OUT*^ and in-strengths *S*^*IN*^ of nodes in GIRCN-RIOT, to be calculated as follow:

$$CAI\left(i\right)=S^{OUT}\left(i\right)=\sum_{j=1}^{N}w_{ij}^{P}$$ (8)

$$CWI\left(i\right)=S^{IN}\left(i\right)=\sum_{j=1}^{N}w_{ji}^{P}$$ (9)

**Fig 12:**
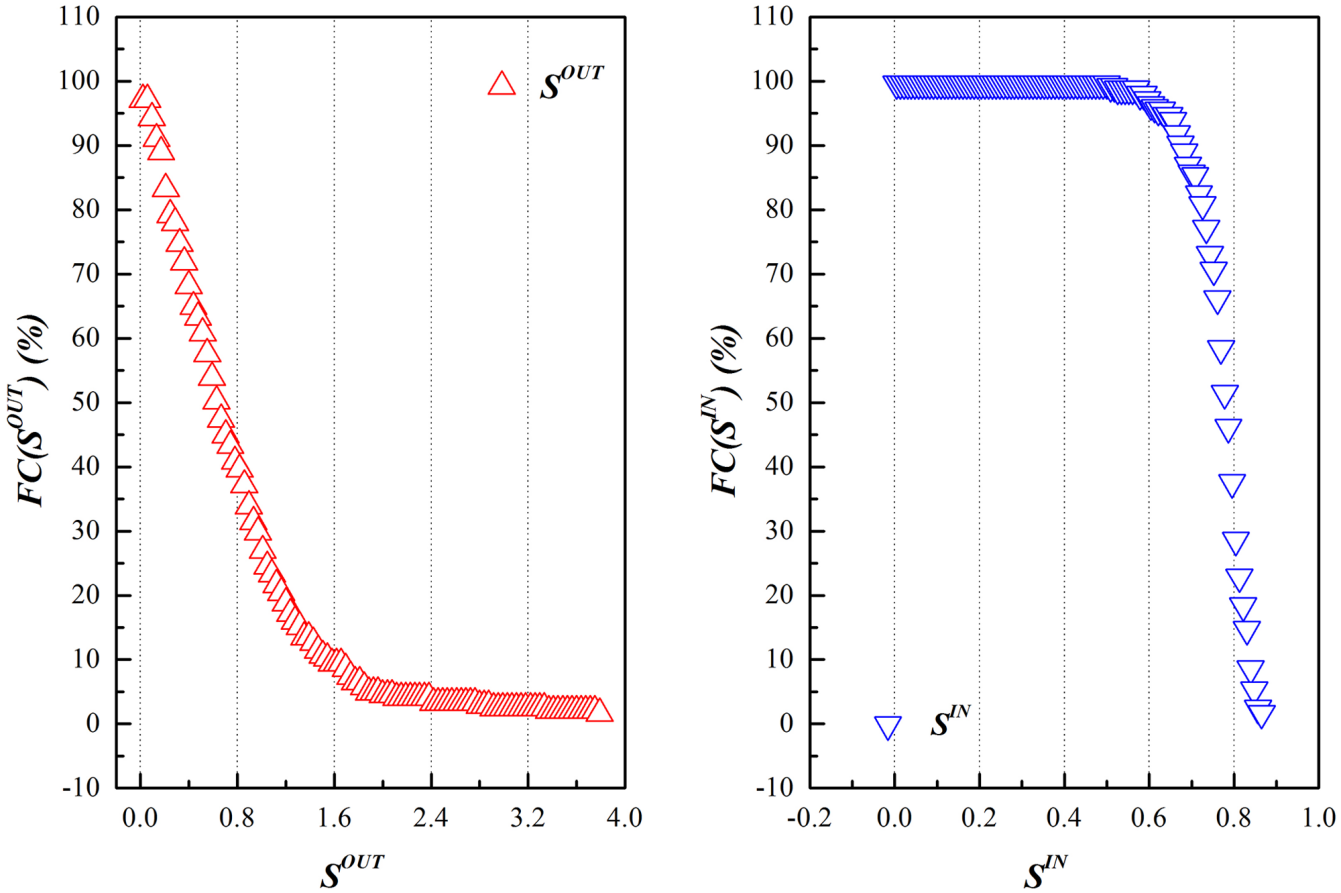
Cumulative distribution of out-strength and in-strength of GIRCN-RIOT-2011.

**Fig 13:**
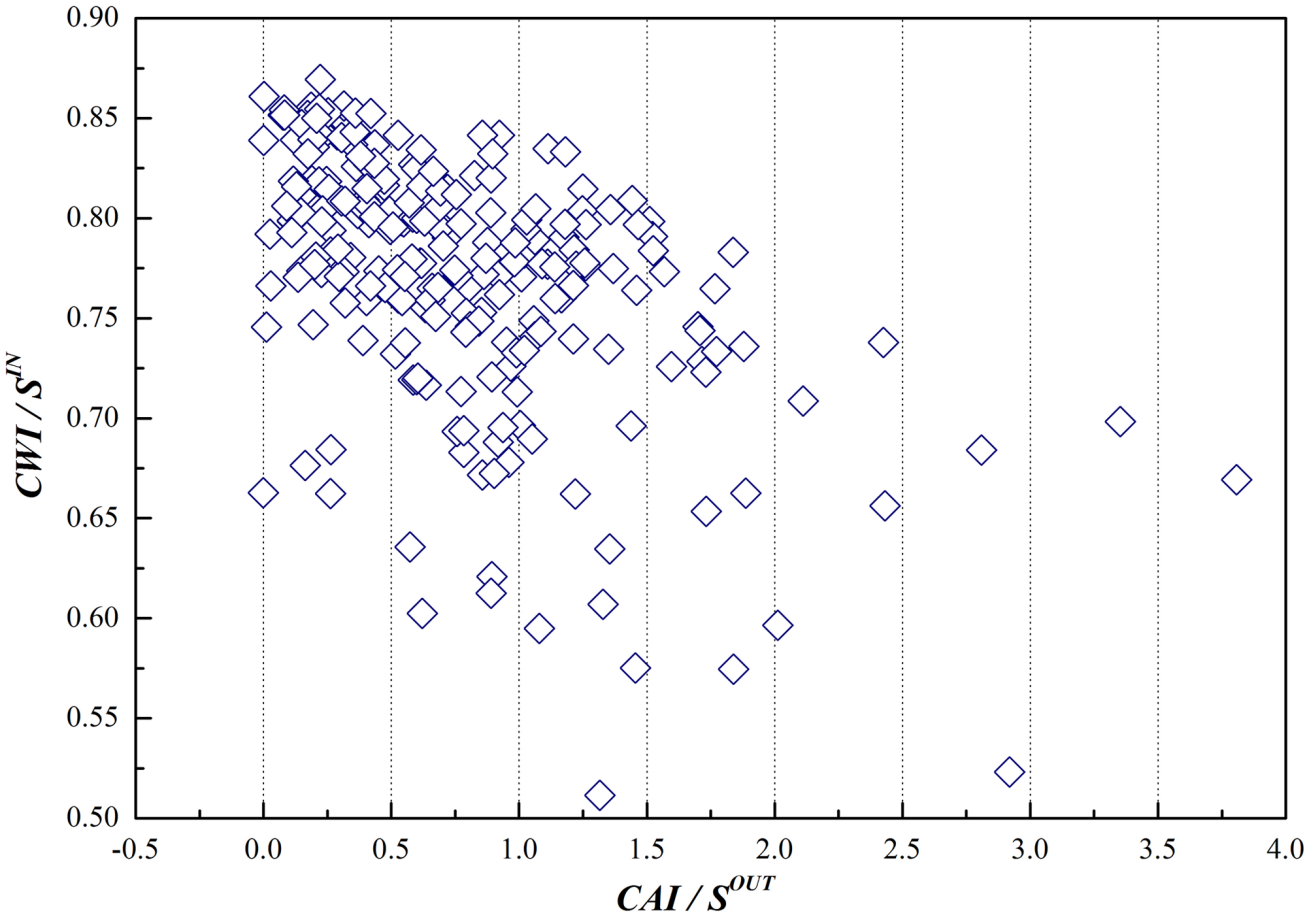
Correlation between CAI and CWI in GIRCN-RIOT-2011.

The concept of node strength covers not only the information of the degree of the node, but also the information of weights of its connecting edges, proving itself to be the integration of local information on the network. CAI and CWI quoted in this paper serve as benchmarks of the competition of industrial sectors on the GVC, taking consideration of both the scale and intensity of competition (cumulative distribution data of out-strength and in-strength is in S2 Table). Cumulative distributions of both out-strength and in-strength are shown in Figs 12 and 13.

Judged from cumulative distribution as shown in Fig 12, there is significant large difference between CAI and CWI, and the former has a more uneven distribution than the later (both CAI and CWI have the even value of 0.755, yet the standard deviation of CAI is 0.594 and that of the CWI is 0.126, and correlation data of CAI and CWI is in S2 Table).

It can be reached from Fig 13 that there is no correlation between CAI and CWI, indicating that they are determined by the structure of GVC and the positions of industrial sectors on it, for there is no necessary connection between the competitive advantages and weaknesses of industrial sectors.

### NCAI and NCWI

On the basis of CAI and CWI, notions of **National Competitive Advantage Index (NCAI)** and **National Competitive Weakness Index (NCWI)** are here introduced as follow:

$$NCAI\left(t\right)=\sum_{i\in \tau \left(t\right)}CAI\left(i\right)$$ (10)

$$NCWI\left(t\right)=\sum_{i\in \tau \left(t\right)}CWI\left(i\right)$$ (11)

where, *t* is the set of economic entities in the RIOT of WIOD, *t* ∈ {*EURO*, *OEURO*, *NAFTA*, *CHN*, *EASIA*, *BRIIAT*, *ROW*}, and *τ* is the set of serial numbers of all industrial sectors in one economic entity of GIRCN-RIOT. Taking China as an example, *τ*(*CHN*) = {106,107,…,140}.

The economic globalization has witnessed that comparative advantage in classical economic theory is not able to fully explain the success and failure of industrial sectors of economic entities in the global environment, and the scholars begin to digest the source and formation of competitive advantage from the prospective of value chain [18]. CAI and CWI proposed in this paper intend to show the competitive statues of industrial sectors on the GVC in the view of econophysics, through evaluation of the strengths among the lower-stream industrial sectors in their competition for the limited supply of intermediates from the upper-stream industrial sectors. In this way, NCAI and NCWI can be indices of economic entity’s competitive strength on the GVC.

### Time series analysis

**Fig 14:**
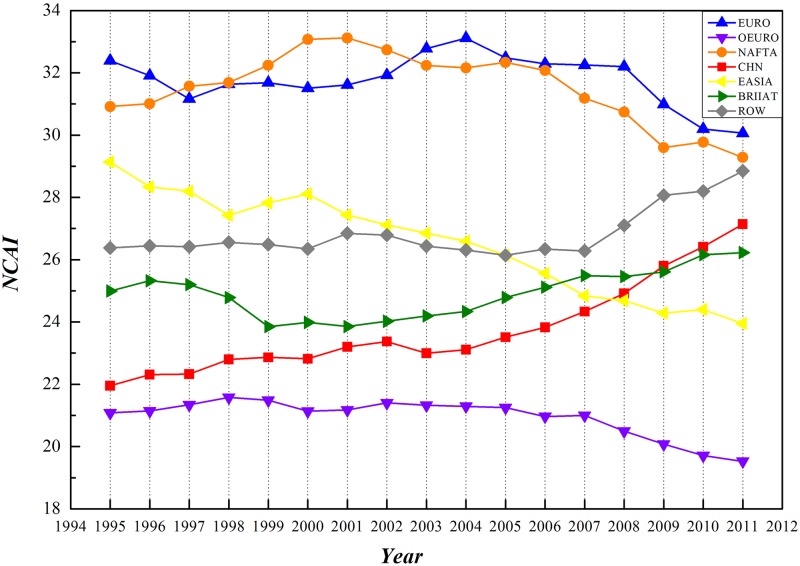
Time-sequential trend of NCAI of economic entities based on GIRCN-RIOT.

**Fig 15:**
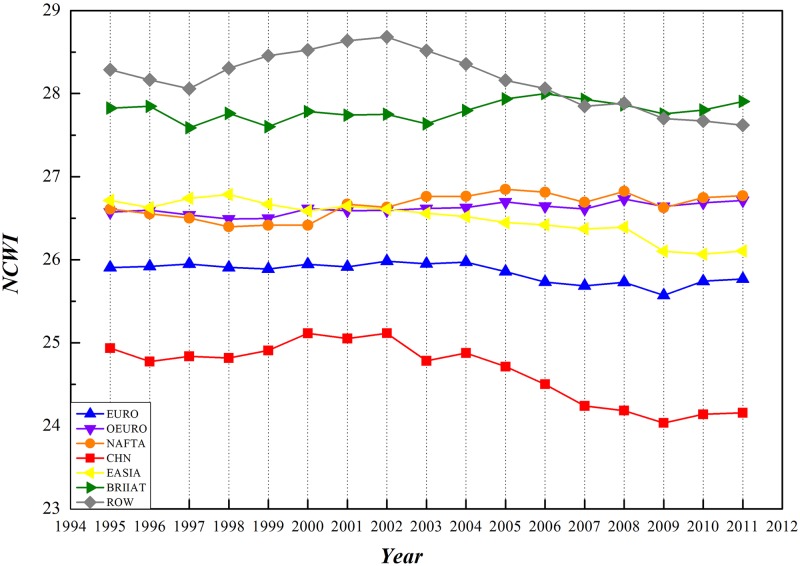
Time-sequential trend of NCWI of economic entities based on GIRCN-RIOT.

WIOD database has provided RIOT data of 1995–2011 covering 17 years. Statistics on NCAI and NCWI of each economic entity have been accomplished based on GIRCN-RIOT models in this paper, reaching a time sequential trend as shown in Figs 14 and 15 (time sequential data of NCAI and NCWI is in S3 Table).

From the prospective of competitive advantages, NCAI of both NAFTA and Eurozone nations have been consistently higher than those of the other nations. With descending after rising, NCAI of NAFTA nations experienced a turning point in 2002 and Eurozone in 2004. NCAI of Eastern Asia and other EU countries have been constantly declining in this period, and have been surpassed by BRIIAT and China in 2006. Both BRIIAT and China have enjoyed a significant rising in NCAI. After 2007, NCAI of the rest of the world had a soaring in NCAI, which will possibly exceed those of NAFTA and even Eurozone nations.

From the prospective of competitive weakness, these economic entities could be in 4 different categories. The rest of the world and BRIIAT nations have the highest in NCWI. NAFTA, other EU, and Easter Asia countries have similar NCWI, belonging to the second group. Eurozone countries contribute to the third group. China has the lowest NCWI, making itself the fourth group.

Taking the above into consideration, it is not hard to see that the integrated competitive advantage of China in the global economic system is continuously improving with decreasing NCWI, embodying a strong competitive power and tremendous potential.

## Simulation

GIVCN-RIOT is able to portray the flows of intermediates between industrial sectors of economic entities on the GVC, and GIRCN-RIOT depicts the competitive relationships among these industrial sectors via RAP. Any disturbance on flows of intermediates among the economic entities would bring influences on the competitive status of relevant entities on the GVC. Static time series analysis on the NCAI and NCWI are first to be carried out in this paper, followed by dynamic simulation on alterations of competitive strengths brought about by changes on trades between China and other economic entities.

### Basic settings

A set of three simulation analysis based on GIVCN-RIOT and GIRCN-RIOT has been done in this paper to realize the impacts of international trade fluctuation on national competitive advantage and weakness, taking the RIOT data in 2011 as the base of the whole simulation application. Taking economic entity X and Y as an example, there exist mainly three kinds of international trade fluctuation in between them: one is X has varied export to Y, one is Y has varied export to X, and another is both X and Y have varied exports to each other. And adjustment has been made to the gross value of one entity’s export to the other in both directions, say from 100% of the basis to 0% (decreasing) and from 100% of the basis to 200% (increasing). Simulations have been made for every 5% of the fluctuation for the calculation of NCAI and NCWI in the GIRCN-RIOT model to reveal the developing trends of NCAI and NCWI under each scenario (all of the simulation data is in S4 Table).

### China vs. NAFTA

**Fig 16:**
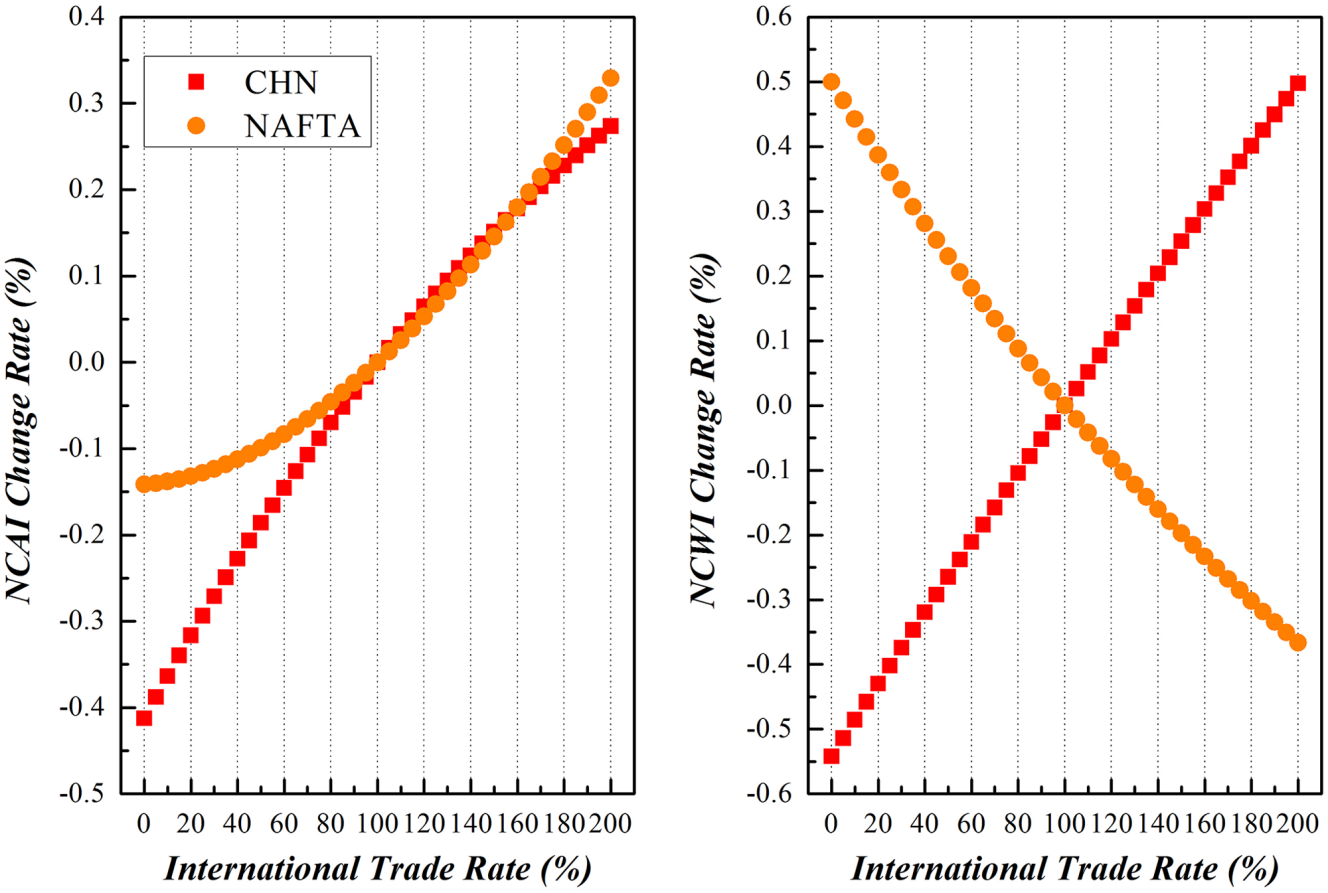
Trends of NCAI and NCWI of China and NAFTA under Scenario (1).

**Fig 17:**
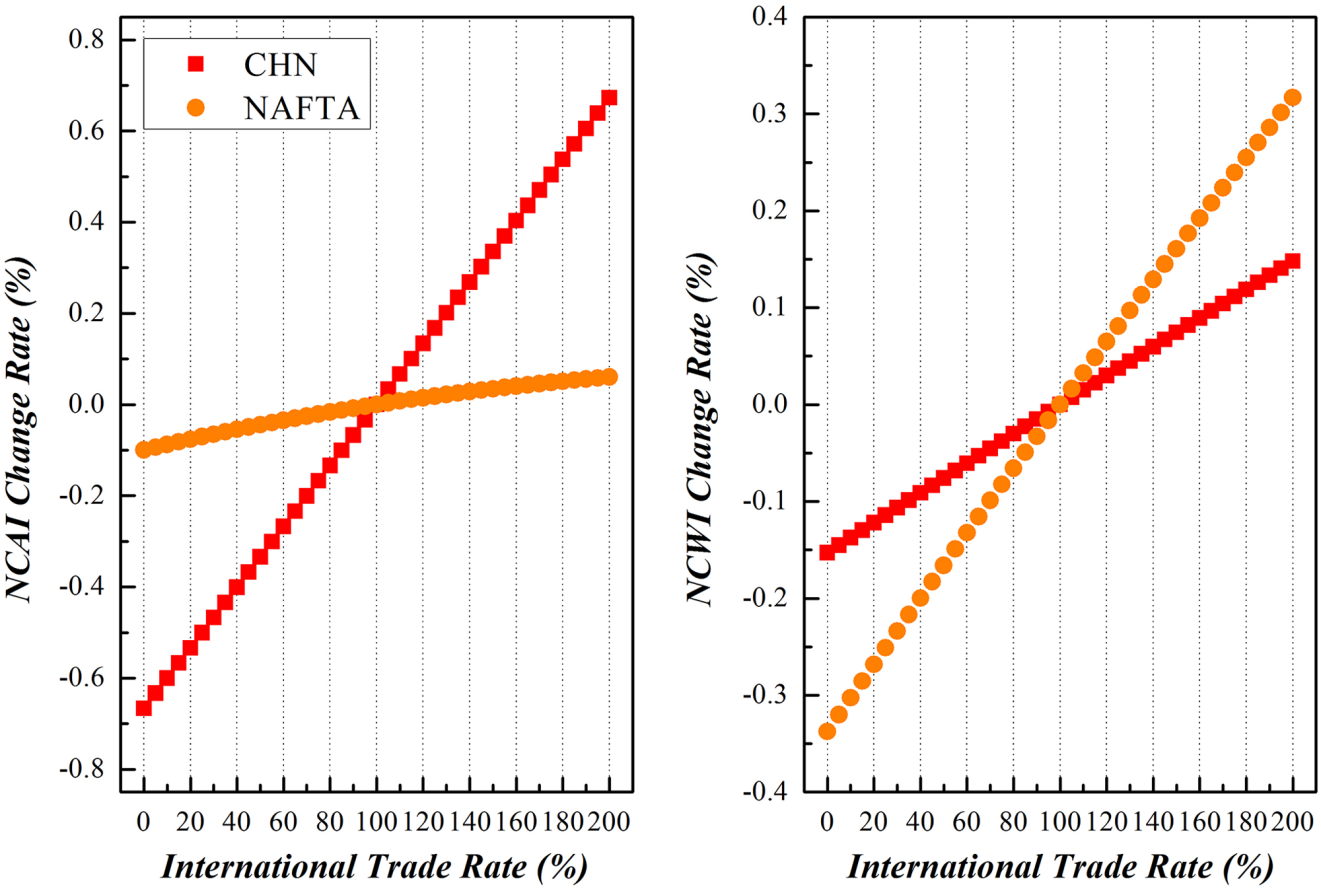
Trends of NCAI and NCWI of NAFTA and China under Scenario (2).

**Fig 18:**
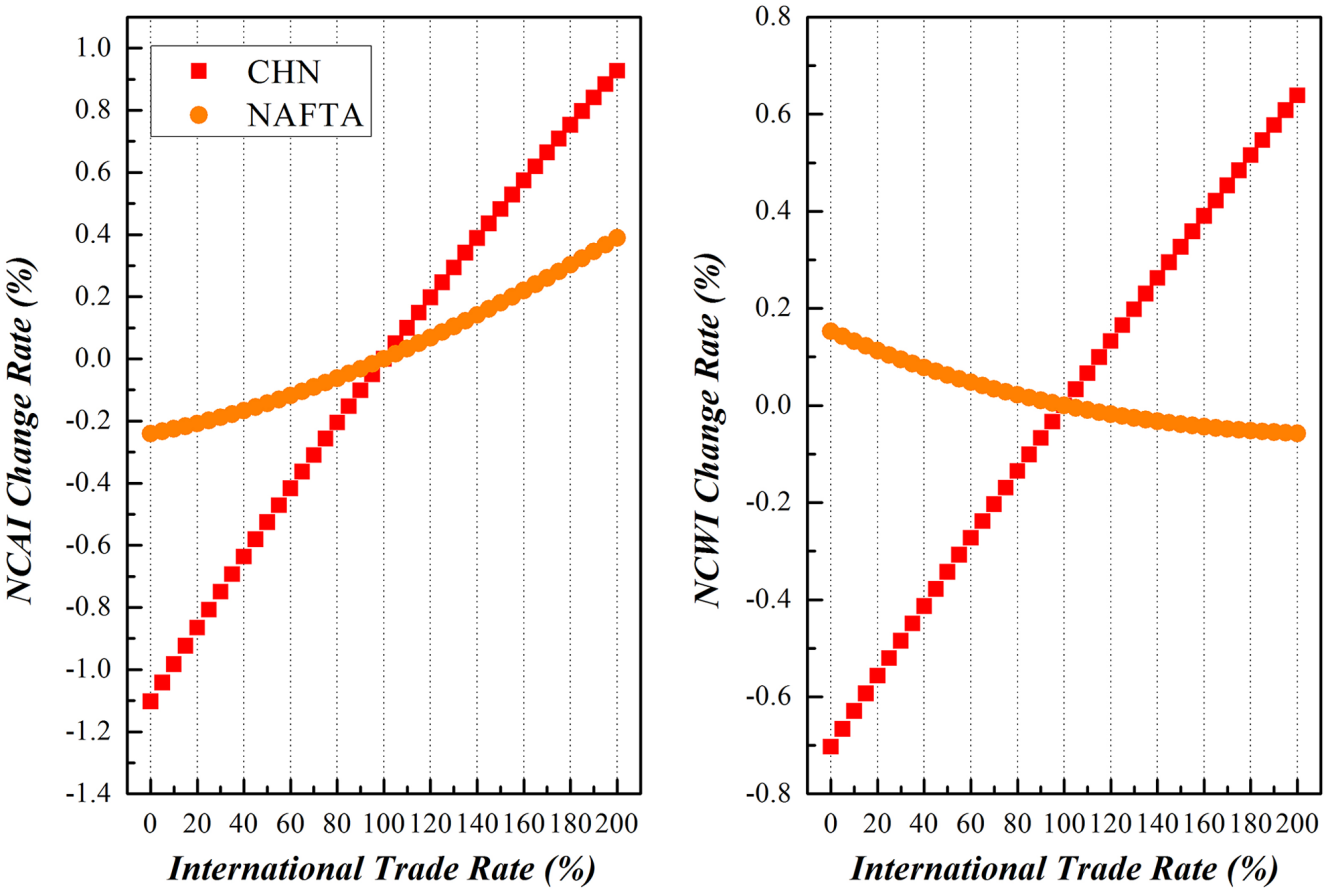
Trends of NCAI and NCWI of NAFTA and China under Scenario (3).

China and NAFTA countries have been chosen as peers to realize the proposed simulation with results shown in Figs 16–18.

Scenario (1) **China varies its export to NAFTA from 0% of its basis to 200%, while NAFTA remains stable**.
  Along with the variation of China’s export to NAFTA from 0% to 200% of the basis, its NCWI is rising more rapidly than NCAI, indicating China will be confronted with more competitive stress while it tries to take a more active part in the globalization. On the other hand, the NCAI of NAFTA increases synchronously with that of China and even surpass when China doubles its export to NAFTA, but its NCWI declines rapidly. This is because that NAFTA depends much on the cheap intermediates as the input supplied from China, and increasing of imports from China will significantly relieve its stress on the GVC.
Scenario (2) **NAFTA varies its export to China from 0% of its basis to 200%, while China remains stable**.
  When the export of intermediates from NAFTA to China increased from 0% to 200% of the basis, the NCAI and NCWI of these two economic entities both rise, but the increase of NCAI of NAFTA is of a larger margin while that of NCWI is of a smaller margin compared with those of China. This brings to the light that China could enhance its competence on the GVC through trade with NAFTA, and NAFTA will be confronted with more competitive pressure from China in the same process. The NCAI of NAFTA has a lower increase rate than its NCWI, and that of China is higher than its NCWI. All these show that scenario is beneficial to China.
Scenario (3) **Both China and NAFTA vary their exports to each other from 0% of the basis to 200%**.
  The basis trend remains similar with that of scenario (1), while both China and NAFTA vary their exports of intermediates to each other from 0% to 200% of the basis. But China has a higher increase of NCAI, indicating that the common increase of trade between the two economic entities will bring more opportunities for China while consolidating the economic position of NAFTA with a relevant lower risk.

### China vs. other economies

**Fig 19:**
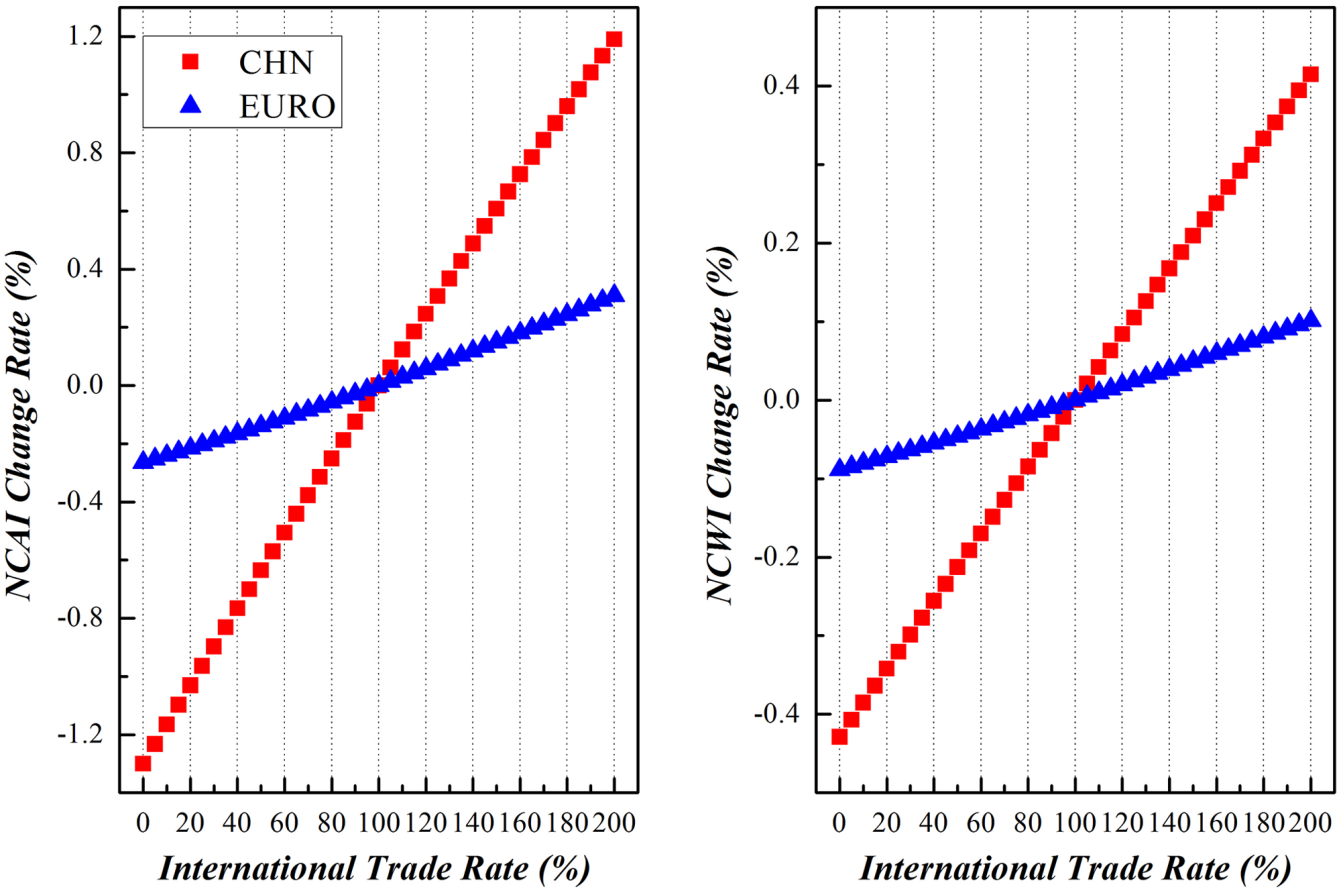
Trends of NCAI and NCWI of Euro-Zone countries and China under Mutual Boosting of Bilateral Trade.

**Fig 20:**
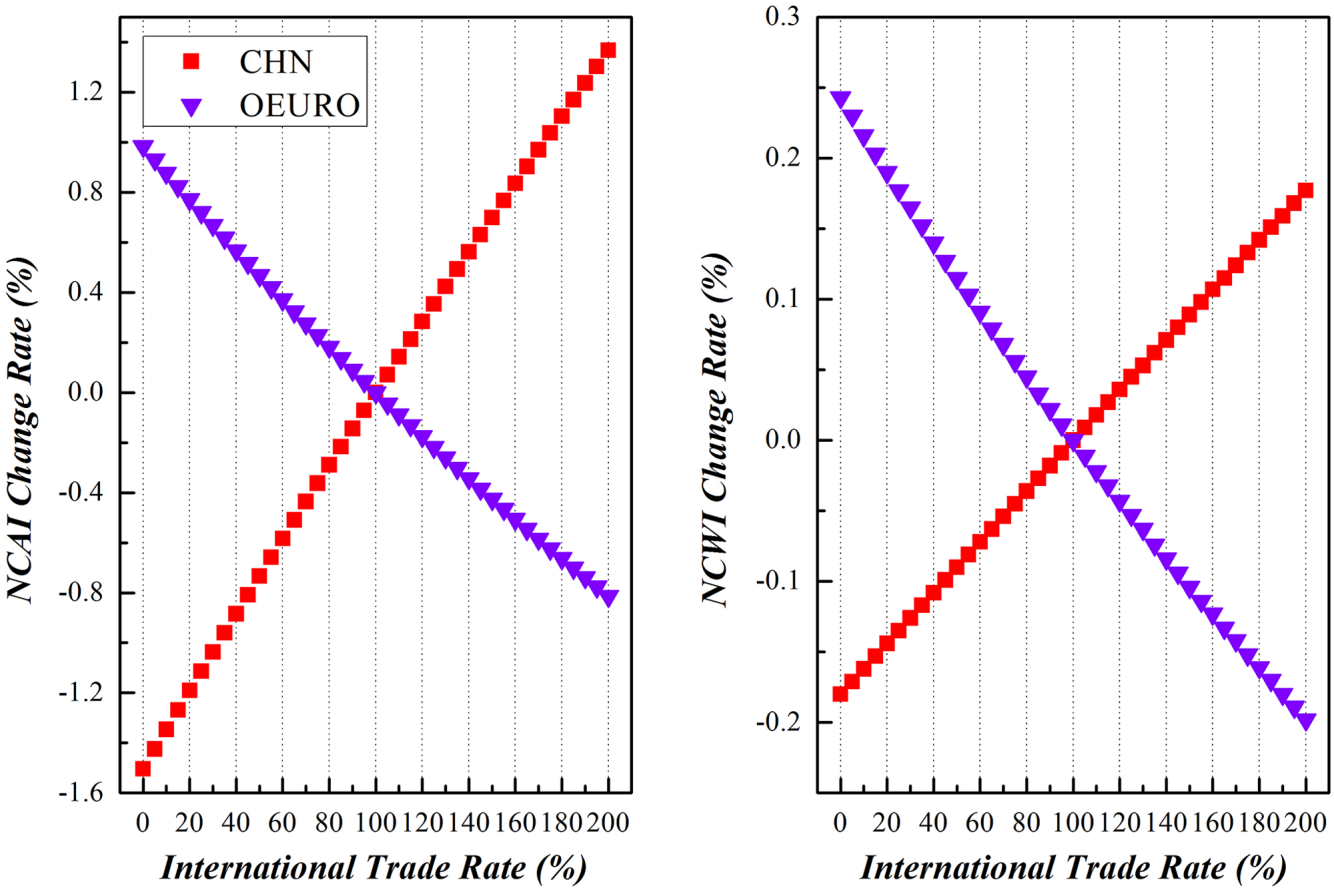
Trends of NCAI and NCWI of other European Union countries and China under Mutual Boosting of Bilateral Trade.

**Fig 21:**
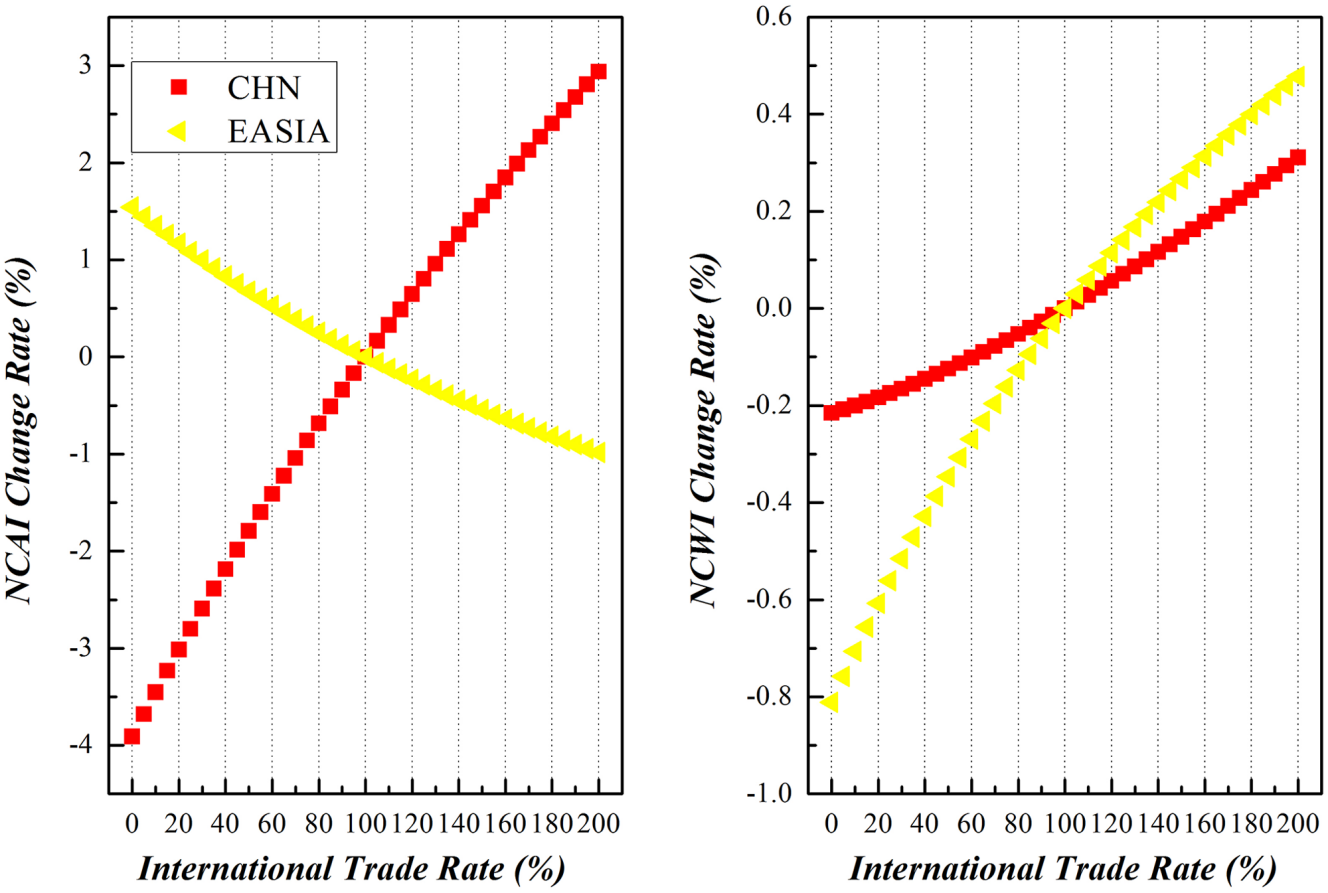
Trends of NCAI and NCWI of Eastern Asian countries and China under Mutual Boosting of Bilateral Trade.

**Fig 22:**
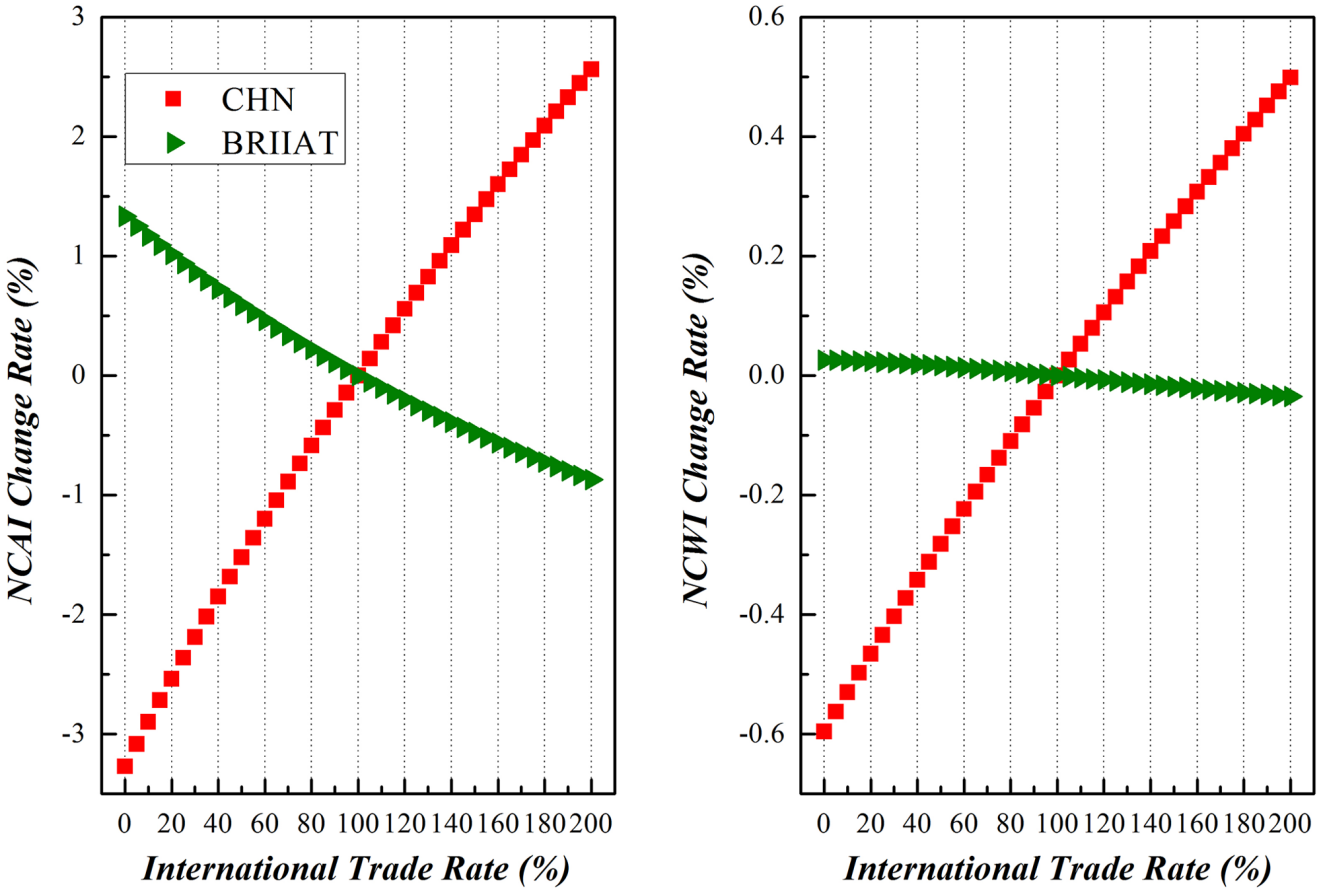
Trends of NCAI and NCWI of BRIIAT countries and China under Mutual Boosting of Bilateral Trade.

**Fig 23:**
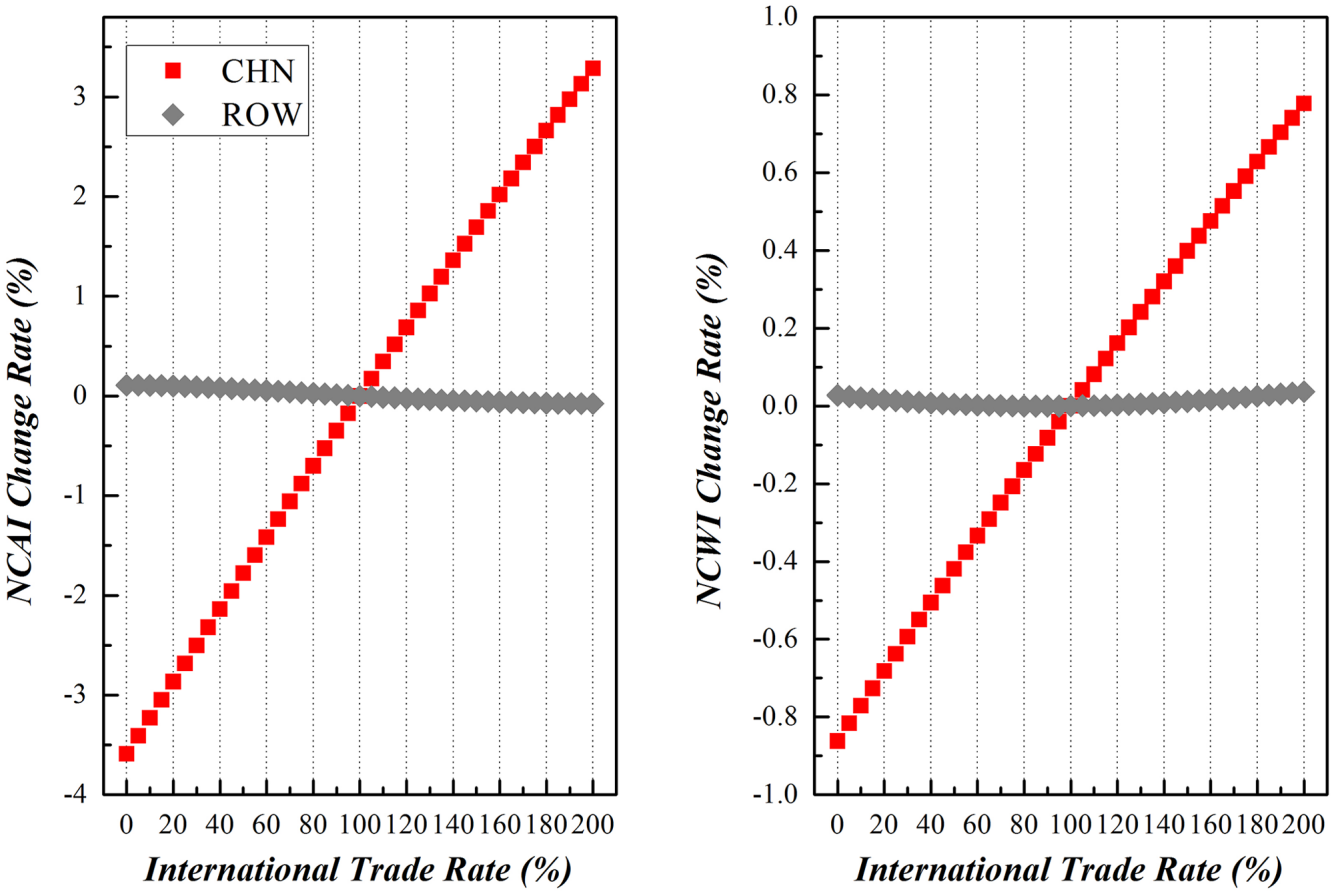
Trends of NCAI and NCWI of other countries and China under Mutual Boosting of Bilateral Trade.

Along with the enforcement of international trade, China and other economic entities around the world would experience different comparative national competitive advantages and/or weaknesses. Due to the limitation of space here, this paper will dedicate to the variation of NCAI and NCWI of China vs other economic entities under scenario (3), as shown in Figs 19–23. Basically speaking, both China’s NCAI and NCWI will increase under most circumstances, but its NCAI has a larger increase rate than NCWI. This shows that strengthening its economic ties with other economic entities is the prerequisite of China’s growing into a trade power, for it will gain more competitive advantages than weaknesses in the process. Nevertheless, other economic entities will incur different impacts in the same process.

Euro countries will have a higher increase of NCAI than NCWI, showing that strengthening their trading partnership with China will bring about more opportunity than the challenge.

Take the trade between China and Germany as an example, it is rather complementary. On one hand, China’s exports to Germany are mainly primary processing and labor-intensive products, such as mechanical and electrical products, textiles and raw materials, furniture / toys / miscellaneous products. On the other hand, its imports from Germany are capital intensive or technology-intensive products, such as mechanical and electrical products, transportation devices.

The difference of trade commodities between the two countries has been determined by the different industrial structures. As an industrialized and developed country, Germany has its industrial structure dominated by its third industries, with add-on values obtained through importing large quantities of primary processing products and exporting technology intensive ones. But China’s industrialization has only lately started. With comparatively less capital accumulation, its industrial structure is dominated by the second industries, calling for imports of massive advanced equipment and technologies. It is clear that further developing trade between China and Euro-Zone countries is mutually beneficial.

Other European Union countries will experience a greater decline of its NCAI than NCWI, showing that their importing of intermediates from China may weaken their economic status in the global market.

On one side, China has a complementary trade with Central and Eastern European countries, importing mainly resources intensive primary products. For instance, China imports copper and its products from Poland, primary products and raw materials from Bulgaria. Yet China’s exports to Central and Eastern European countries are mainly manufactured goods. So enhanced trade with Central and Eastern European countries will bring diversification to China’s trade structure, on top of providing the raw material for China’s developing economy.

On the other side, China has a competitive exporting market and commodity structure with Central and Eastern European countries. For instance, Poland, Czech, and Hungary have similar trade commodity structure with China of exporting mechanical and electrical products to Euro-Zone countries. But China has obviously comparative labor cost advantage, and thus more intensive specification in globalization than Central and Eastern European countries. In this way, China enjoys more competitive advantage in exporting to Euro-Zone countries, bringing about negative impacts on the comparative competitive advantages of other European Union countries.

The declining of NCAI and rising of NCWI of Eastern Asian countries shows that their bilateral developing trade between China will weaken their competitive advantages while worsen their competitive weakness. And this is actually their motive in joining TPP.

The nature of trade between China and Eastern Asian countries is the “triangle model” that China imports intermediates from Eastern Asian countries for further processing before finally exporting to American and European countries as final products. So China has played a pivotal role in the manufacturing network of China and Eastern Asian countries. The mutual promotion of bilateral trade between the two economic entities will boost the competitive strength of China in GVC and attenuate the competitive advantage of Eastern Asian countries. Further exporting from China might seize the Eastern Asian markets, while the competitive abilities of their industrial sectors are to abate, and aggrandize their competitive weaknesses.

There is more declining of NCAI than NCWI of the BRIIAT countries under this circumstance, showing that their weakness outweighs possible gains in their trade with China. With comparative advanced manufacturing industries and technologies, China enjoys an obvious comparative advantage is their trade with BRIIAT countries.

The abundance of natural resources of BRIIAT countries guarantees their advantages in primary products, which is the ultimate demand of China in the trade. For instance, China imports from Brazil primary products such as soybeans, oil, and iron ore, from India resource products such as mineral products, cotton products, and copper products, from Australia great quantities of iron ores, from Russia manufactured goods (mostly labor intensive), energy and resource intensive products.

The above-mentioned properties will enhance the complementation between these two economic entities with the more distinctive global specification. But the international trade structure like these enables China to be ahead of the others in the BRIIAT markets. On the other hand, export from BRIIAT is mainly primary products of an upper stream on the GVC, making it gain a comparatively small portion of add-on value in the developing bilateral trade, with relatively little promotion of their competitive weakness.

Trade with other countries with China is of limited scale, so any destabilization will bring a little variation on the NCAI and NCWI of both parties.

## Conclusions

How to reproduce the topological structure of the global economic system from the perspective of system science and excavate its operation law has been a major problem that puzzles the academia for a long time. With the research framework based on physical economics and complex network theory, this paper analyzes the input-output relationship of intermediates among major economic entities between 1995–2011 with RIOT data from WIOD, and extract the competitive relations among them with RAP. Further introduction of four indices of CAI, CWI, NCAI, and NCWI is to reveal the competitive status of industry sectors and economies on the GVC. The contribution of the paper is as follow:

**(1) Construction of GIVCN-RIOT model based on ICIO data from WIOD to reproduce the topological structure of the global economic system**.
  The adoption of ICIO data as resources in this paper is not only for its ability of reshowing flows of intermediate products, final products and, services but also for possible comparison on the same basis. The proposed infrastructure of ICIO networks based on econophysics can focus on the topological structure of GVC on top of partial analysis on international trades. Further mining of network structural characteristics in this way can reveal the functional properties of countries and their industry sectors, enhancing the accuracy of analysis on the GVC.
**(2) Extraction of competitive relations on scarce resources on the GVC among economic entities and their industry sectors based on Resource Allocation Process**.
  Having consideration of resource scarcity, this paper uses bipartite graphs to modify the GIVCN-RIOT model, distinguishing the simultaneous roles of industry sectors on the GVC as upper-stream and lower-stream ones, by constructing GIVCN-RIOT-BIPARTITE model. Mapping this bipartite graph into the direction of participants (lower-stream industry sectors) leads to revised GIRCN-RIOT model. This will give hints on competition of any industry sector of lower-stream status with the others, which is a breakthrough in traditional IO analysis.
**(3) Simulation of competitive advantages and weaknesses of economic entities based on network structural measurements**.
  GIRCN-RIOT depicts competitions among economic entities and their industry sectors. The directions of its edges show the differences of competitive relations, with the out-strengths and in-strengths revealing their competitive advantages and weaknesses on the GVC. Further simulations have been made here on various scenarios of trades among economic entities for observation of trends of their competitive advantages and weaknesses on the GVC, to reveal the evolution mechanics of international trades from the prospective of econophysics.

## Supporting information

S1 Table Detailed names and abbreviations of sectors in RIOT of WIOD. (XLSX)

S2 Table Cumulative distribution data of out-strength and in-strength and correlation data of CAI and CWI. (OPJ)

S3 Table Time sequential data of NCAI and NCWI. (OPJ)

S4 Table Results of simulation. (OPJ)
